# A Higher-Yielding Route for the Synthesis of Molecular Precursors to Thin Films of Gold by Atomic Layer Deposition

**DOI:** 10.1021/acs.organomet.5c00242

**Published:** 2025-09-16

**Authors:** Ryan K. Brown, Joseph N. Bunyan, Ashi Agrawal, Graeme Hogarth, Christopher S. Blackman, Leonardo Santoni, Eva Rimoldi Rudatis, Caroline E. Knapp, David Pugh

**Affiliations:** a Department of Chemistry, 4616 King’s College London, Britannia House, 7 Trinity Street, London SE1 1DB, U.K.; b Department of Chemistry, 4919 University College London, 20 Gordon Street, London WC1H 0AJ, U.K.

## Abstract

A synthetically straightforward and high-yielding route to the series of complexes [AuMe_2_(dtc)] (dtc = dithiocarbamate) has been developed using dialkyl-, cyclic-dialkyl-, and diaryldithiocarbamate ligands. These compounds were screened by thermogravimetric analysis (TGA) to determine their viability as molecular precursors to thin films of elemental gold by atomic layer deposition. TGA revealed that the volatility of the precursor decreased as the mass of the precursor increased, making the heavier molecules less suitable precursors. However, [AuMe_2_(Me_2_dtc)], the lightest member of the series, is potentially a better precursor than [AuMe_2_(Et_2_dtc)] due to its higher volatility and slightly lower residual mass.

## Graphical abstract

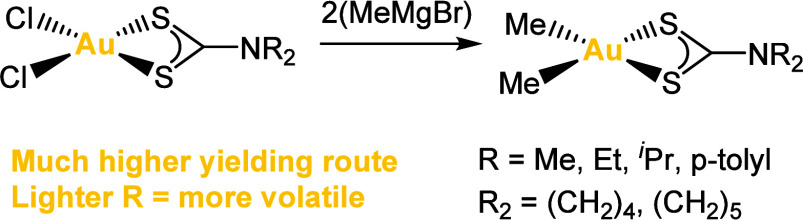

## Introduction

Thin films of metallic gold have been used as decorative coatings on artwork for millennia. Modern applications of thin gold films utilize the advantageous physical properties of gold such as high electrical conductivity, high thermal conductivity, and resistance to corrosion.^1^ Despite considerable success using chemical vapor deposition (CVD) to deposit thin films of gold,^2^ the first successful atomic layer deposition (ALD) of gold thin films was only reported in 2016 using [AuMe_3_(PMe_3_)] as a precursor in the presence of water and oxygen plasma.^3^ The same precursor was used in 2019 to deposit gold films by ALD with hydrogen plasma,^4^ and a subsequent report investigated the deposition behavior of [AuMe_3_(PMe_3_)] on hydrogen-passivated surfaces.^5^ The gold­(I) precursor [AuCl­(PEt_3_)] was used to deposit gold films by ALD in combination with 1,4-bis­(trimethylgermyl)-1,4-dihydropyrazine as a reducing agent,^6^ and recently, a gold­(III) dialkyldithiophosphate (Figure 1) was used to deposit gold thin films by both CVD and ALD.^7^

**Figure 1:**
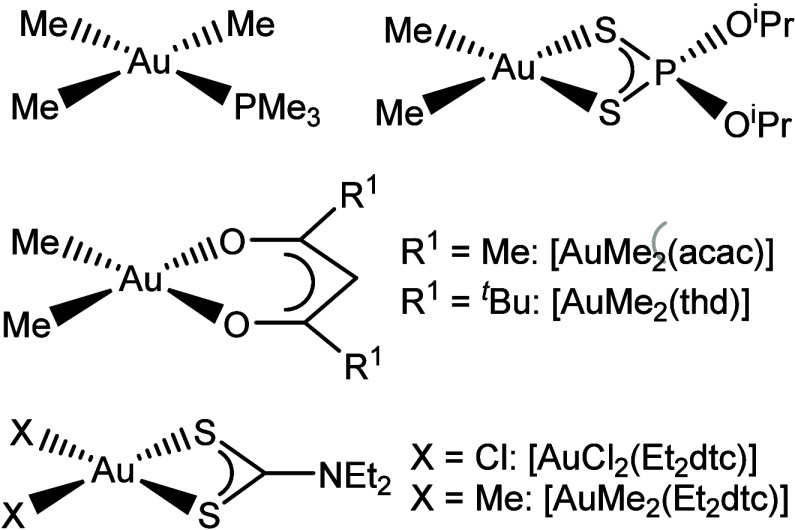
Existing precursors to Au thin films via ALD: [AuMe_3_(PMe_3_)] (top left), [AuMe_2_(S_2_P­(O^*i*^Pr)_2_)] (top right), [AuMe_2_(bdk)] (middle), and [AuX_2_(Et_2_dtc)] (bottom).

In 2017, Mäkelä et al. conducted a small screen of molecular Au­(III) complexes using thermogravimetric analysis (TGA), specifically looking for viable ALD precursors.^8^ [AuMe_2_(Et_2_dtc)] (Et_2_dtc = diethyldithiocarbamate) exhibited the best combination of volatility and thermal stability when compared to precursors such as “[AuCl_2_(Et_2_dtc)]”, [Au­(μ_2_-OAc)­Me_2_]_2_, and “AuCl_3_”.^9^ This was consistent with an earlier study, which compared [AuMe_2_(Et_2_dtc)] to [AuMe_2_(bdk)] (bdk = β-diketonate: acetylacetonate and 2,2,6,6-tetramethylheptanedionate) using TGA.^10^ [AuMe_2_(Et_2_dtc)] exhibited a single-step mass loss at ∼240 °C with very little residual mass, indicating high volatility but also good thermal stability. Recent reviews of molecular precursors for the CVD and ALD of gold films suggest that [AuMe_2_(dtc)]-type compounds are a promising class of molecular precursors, but there have been no screening studies which systematically varied the dtc ligand.^11–14^

Only a handful of [AuMe_2_(dtc)] complexes have been previously synthesized (Figure 2). The most common examples are [AuMe_2_(Me_2_dtc)] and [AuMe_2_(Et_2_dtc)], where the addition of M­(dtc) (M = Li and Na) to isolated [AuBrMe_2_]_2_ or [AuIMe_2_]_2_ afforded the desired product.^15–18^ [AuMe_2_(Me_2_dtc)] was also synthesized by oxidative addition of Na­(Me_2_dtc)/Br_2_,^19^ or tetramethylthiuram disulfide,^20,21^ to [AuMe­(PPh_3_)]. Examples of [AuMe_2_(dtc)] where the dtc ligand had longer alkyl chains (^*n*^Pr, ^*n*^Bu, and ^*n*^nonyl), as well as Me and Et, were reported by van der Kerk and co-workers in 1964.^22^ They also used the [AuIMe_2_]_2_ route, but the gold synthon was generated *in situ* and not isolated. Notably, they also studied the reaction of “[AuCl_2_(dtc)]” with CdMe_2_, which also afforded the desired [AuMe_2_(dtc)] products. No examples of [AuMe_2_(dtc)] with diaryldithiocarbamate ligands are known, but the Na^+^ salt of *N*-methyl-*N*-phenyldithiocarbamate was reacted with [AuIMe_2_]_2_ affording [AuMe_2_(MePhdtc)].^23^ Finally, [AuMe_2_(pip-dtc)] (pip-dtc = piperidinyl dithiocarbamate) was included in a patent, although no synthetic or analytical data for this compound were disclosed.^24^

**Figure 2:**
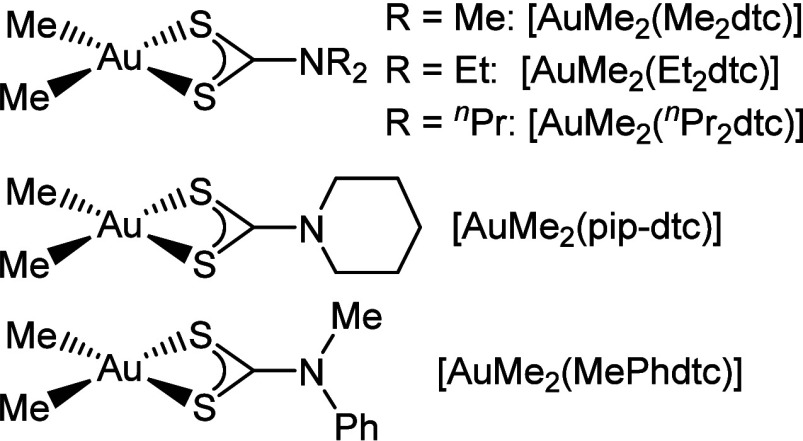
Literature examples of [AuMe_2_(dtc)] complexes.

None of the [AuMe_2_(dtc)] compounds have been synthesized in a high yield from commercially available “AuCl_3_”. This is primarily due to the synthesis of [AuIMe_2_]_2_ (Scheme 1, top), which affords the desired intermediate in only moderate yield. This means that the route is somewhat wasteful and expensive even though the subsequent salt metathesis reactions with Na­(dtc) are essentially quantitative.^25^ Given the high price of gold, as well as the need to reduce waste and increase sustainability, a better route to compounds of the type [AuMe_2_(dtc)] was needed. Herein, we report a higher-yielding and synthetically easier route to access [AuMe_2_(Et_2_dtc)] (Scheme 1, bottom), as well as the synthesis and characterization of several novel [AuMe_2_(dtc)] analogues, which are screened by TGA to ascertain their suitability for the ALD of gold thin films.

**Scheme 1:**
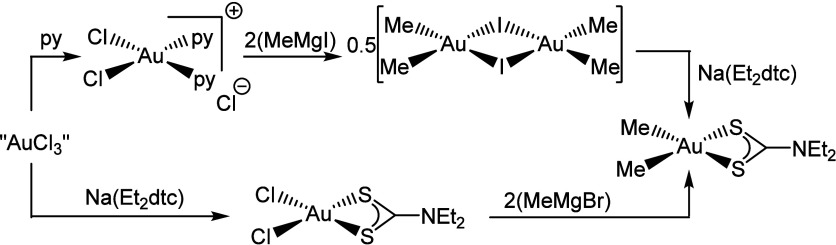
Literature Synthesis of [AuMe_2_(Et_2_dtc)] (Top) and Our Improved Route (Bottom)

## Results and Discussion: Precursor Synthesis

The series of compounds [AuX_2_(dtc)] (X = halide) has been known since the 1960s. For decades, the generally accepted understanding of the synthesis of these compounds was that adding one molar equivalent of M­(dtc) (M = Li^+^, Na^+^, etc.) to a solution of “AuX_3_” (X = Cl and Br) led to the sole formation of [AuX_2_(dtc)], whereas using two molar equivalents of M­(dtc) resulted in the sole formation of the ion pair [Au­(dtc)_2_]­X.^26^ It is now known that this is incorrect: the [AuX_2_(dtc)] system exists in a solution-phase equilibrium with [Au­(dtc)_2_]­[AuX_4_], and it is not possible to obtain [AuX_2_(dtc)] as a single product in solution.^27,28^ However, through careful tuning of the synthetic conditions, it is possible to generate a mixture where [AuX_2_(dtc)] is the majority component (e.g., an ∼17:1 ratio for dtc = Et_2_dtc), which can be used in further syntheses. It is also possible to fractionally crystallize pure [AuX_2_(dtc)] although the equilibrium resumes as soon as the crystals are redissolved in an organic solvent.^27^

Since the crystallization of pure [AuCl_2_(Et_2_dtc)] is slow and not amenable to scale, an initial study was conducted by reacting MeMgBr with the “[AuCl_2_(Et_2_dtc)]” mixture using different ratios of a neutral compound to salt. Initially, methylation of a mixture comprising an ∼3:5 ratio of [AuCl_2_(Et_2_dtc)]:[Au­(Et_2_dtc)_2_]­[AuCl_4_] (obtained from the aqueous synthesis of “[AuCl_2_(Et_2_dtc)]” reported by Nilakantan et al.)^29^ resulted in 54% isolated yield of [AuMe_2_(Et_2_dtc)]. This reaction also generated a significant amount of black solid, likely formed from the reaction of [Au­(Et_2_dtc)_2_]­[AuCl_4_] with MeMgBr followed by decomposition to metallic gold. However, methylation of a “[AuCl_2_(Et_2_dtc)]” mixture with an ∼17:1 ratio of [AuCl_2_(Et_2_dtc)]:[Au­(Et_2_dtc)_2_]­[AuCl_4_][Bibr ref27] led to the isolation of [AuMe_2_(Et_2_dtc)] in 88% yield. Finally, use of pure, crystalline [AuCl_2_(Et_2_dtc)] as a starting material did result in a slight increase in isolated yield of [AuMe_2_(Et_2_dtc)] to 91%. In all cases, the desired organometallic [AuMe_2_(Et_2_dtc)] was isolated as a single pure product, and no evidence of solution-phase equilibration was seen.

As a result of this screen, it was decided that the marginal gain in isolated yield obtained by using crystalline [AuCl_2_(Et_2_dtc)] did not justify the time expended on the crystallization process. Hence, all subsequent methylation reactions (below) were conducted using a “[AuCl_2_(dtc)]” mixture, which had been synthesized in refluxing acetone for 16 h.^27^ The outcome was that the overall yield of [AuMe_2_(Et_2_dtc)] starting from commercially obtained AuCl_3_ (Scheme 1, bottom) was significantly improved to 78% compared to the existing route via [AuIMe_2_]_2_ (Scheme 1, top). The new synthetic route was also quicker, with analytically pure samples of [AuMe_2_(dtc)] obtained in <24 h starting from commercially available “AuCl_3_”.

With this result in hand, we turned our attention to the synthesis of a series of compounds [AuMe_2_(dtc)] using a variety of different dtc ligands. The reaction of a “[AuCl_2_(Me_2_dtc)]” mixture (where [AuCl_2_(Me_2_dtc)] was the dominant component) with MeMgBr led to the isolation of [AuMe_2_(Me_2_dtc)] in 79% yield. The ^1^H NMR spectrum of the product contained the expected two singlets, with the Au–Me signal at ∼1 ppm. No evidence of side products or other impurities was seen, and elemental analysis confirmed that the desired product had been synthesized. Recrystallization from warm CH_2_Cl_2_ resulted in the growth of X-ray quality crystals of [AuMe_2_(Me_2_dtc)] (Figure 3).

**Figure 3:**
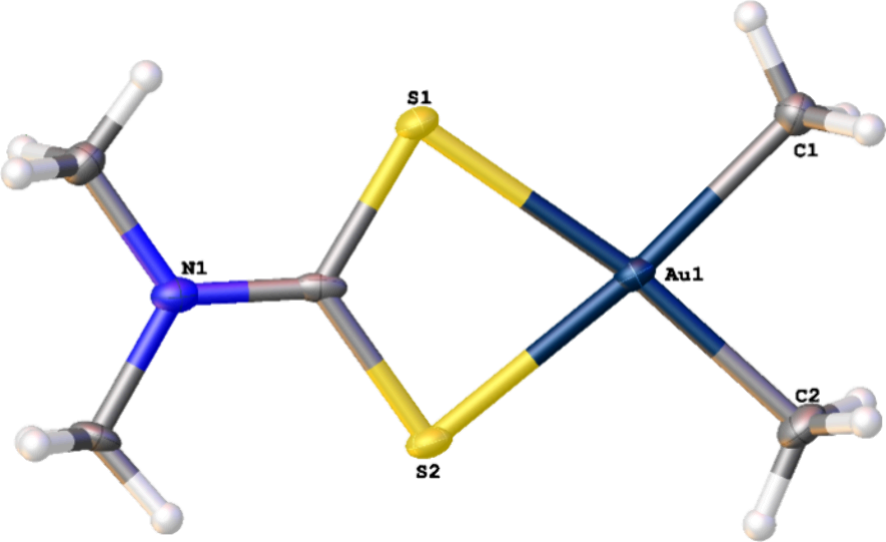
Solid-state structure of [AuMe_2_(Me_2_dtc)] with thermal ellipsoids at the 50% probability level. Selected bond lengths (Å): Au–S1, 2.415(1); Au–S2, 2.4092(11); Au–C1, 2.066(4); Au–C2, 2.059(4).

[AuMe_2_(Me_2_dtc)] crystallized in the triclinic space group $P\overset{-}{1}$ as a four-coordinate slightly distorted square planar Au­(III) complex. Au–C and Au–S bond lengths were consistent with those found in the structure of [AuMe_2_(Et_2_dtc)],^17^ and the molecule is planar: the RMS deviation of the non-H atoms from the plane defined by all nine non-H atoms is only 0.015 Å. The extended structure shows no evidence of aurophilic interactions (shortest Au···Au distance = 5.48 Å); instead, intermolecular CH···Au interactions between Au and one of the *N*-methyl groups on a neighboring dtc ligand are present. The CH···Au distance of 3.040 Å is ∼0.5 Å less than the sum of van der Waals radii for Au and H^30^ and consistent with stated literature examples of CH···Au­(III) hydrogen bonding.^31^ The concept of CH···Au­(III) hydrogen bonding is still controversial,^31–33^ but the CH···Au angle in [AuMe_2_(Me_2_dtc)] approaches linearity (171.60°) indicating strong directionality to this interaction, something which existing literature examples do not exhibit.

The diisopropyldithiocarbamate analogue [AuMe_2_(^*i*^Pr_2_dtc)] was synthesized in a similar manner, although this compound was isolated as a soft, sticky solid, which resisted attempts at crystallization. One attempt did generate a small amount of tiny colorless crystals, but single-crystal X-ray analysis of these revealed that they were [Au­(^*i*^Pr_2_dtc)_2_]­Cl·H_2_O, a byproduct left over from the synthesis of “[AuCl_2_(^*i*^Pr_2_dtc)]” (see the SI for further details). A significant amount of a silicon grease contaminant remained in samples which were synthesized under standard Schlenk conditions (i.e., with use of vacuum grease on ground glass joints) despite multiple washes with hydrocarbon solvents, but this contamination could be avoided by conducting the methylation reaction in a greaseless environment followed by a standard aqueous workup in air.

The ^1^H NMR spectrum of [AuMe_2_(^*i*^Pr_2_dtc)] contained very broad resonances for the ^*i*^Pr_2_dtc ligand, which did not resolve even upon cooling to −50 °C, although the Au–Me peak at ∼1 ppm was a sharp singlet at all temperatures. This is likely due to a facile rotation of one of the ^*i*^Pr groups about the C–N bond: the crystal structures of both Na­(^*i*^Pr_2_dtc)^34^ and [AuCl_2_(^*i*^Pr_2_dtc)] ^27,35^ contained a single intramolecular CH···S hydrogen bond between the methine CH of one ^*i*^Pr group and a sulfur atom on the same dtc ligand, along with broad ^1^H NMR spectra. It is also noteworthy that the rotation of an ^*i*^Pr group has been observed to take place within single crystals of [Ni­(^*i*^Pr_2_dtc)_2_] at RT, which manifested as thermosalience on a macroscale.^36^ A satisfactory elemental analysis confirmed that [AuMe_2_(^*i*^Pr_2_dtc)] had been synthesized.

Dithiocarbamate ligands based on the cyclic amines pyrrolidine and piperidine were also investigated. The reaction of MeMgBr with a mixture of “[AuCl_2_(pyrr-dtc)]” or “[AuCl_2_(pip-dtc)]” (where [AuCl_2_(dtc)] was the dominant component) led to the isolation of [AuMe_2_(pyrr-dtc)] and [AuMe_2_(pip-dtc)], respectively, in good yields. The ^1^H NMR spectra of each compound were consistent with the expected structures, with the expected Au–Me peaks at ∼1 ppm and complex multiplets for the methylene groups within the rings. Satisfactory elemental analyses were also obtained. Crystals were grown from a warm CH_2_Cl_2_ solution, and the structures of [AuMe_2_(pyrr-dtc)] (Figure 4a) and [AuMe_2_(pip-dtc)] (Figure 4b) were obtained.

**Figure 4:**
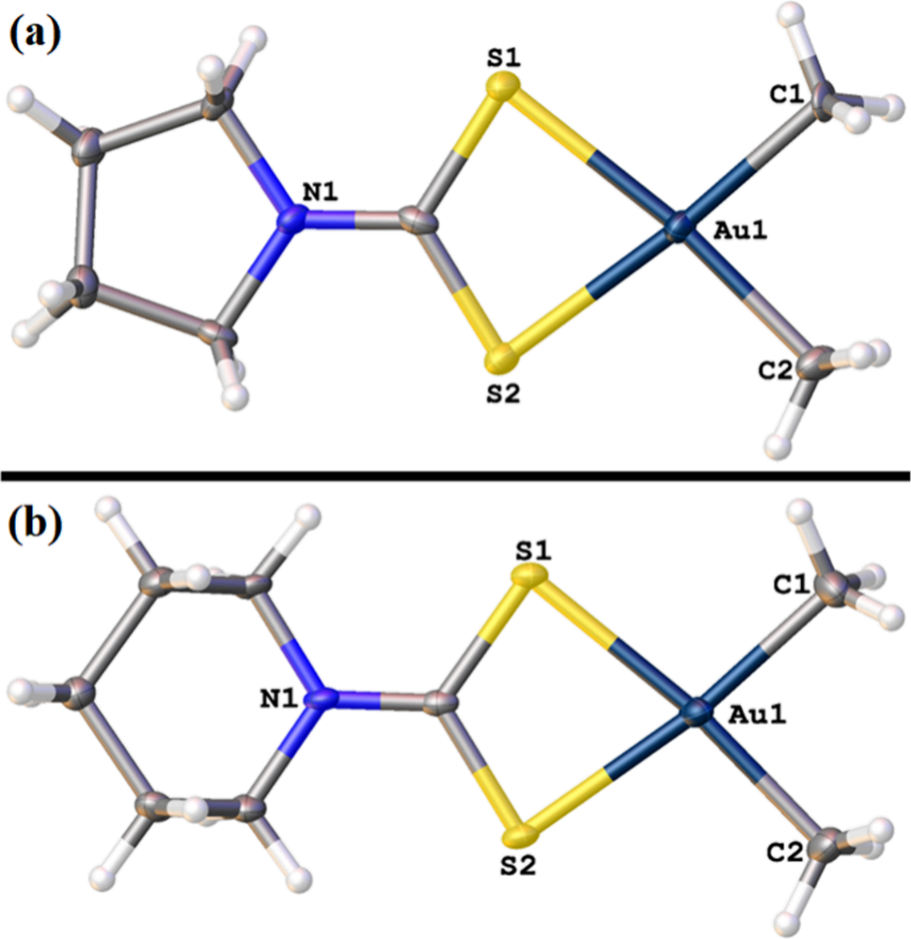
Solid-state structures of (a) [AuMe_2_(pyrr-dtc)] with thermal ellipsoids at the 50% probability level. Selected bond lengths (Å): Au–S1, 2.416(2); Au–S2, 2.406(2); Au–C1, 2.091(9); Au–C2, 2.064(9). (b) [AuMe_2_(pip-dtc)] with thermal ellipsoids at the 50% probability level. Selected bond lengths (Å): Au–S1, 2.4068(7); Au–S2, 2.4006(7); Au–C1, 2.064(3); Au–C2, 2.073(3).

Both molecules contain slightly distorted square planar d^8^ Au­(III) centers. The Au–C bond lengths in both structures were within a narrow range of 2.064–2.091 Å, which is consistent with the Au–C bond lengths observed for [AuMe_2_(Me_2_dtc)] and [AuMe_2_(Et_2_dtc)].^17^ A similar trend was observed for the Au–S bond lengths in both molecules. No aurophilic interactions were observed in either structure, with the shortest intermolecular Au···Au distance being 5.5 Å for [AuMe_2_(pip-dtc)].

Diaryl dithiocarbamates are less common than their dialkyl (or cyclic-dialkyl) analogues, although recent investigations have shown that they can be used as ligands in single-source precursors for metal sulfide materials.^37–39^ We recently reported that the “[AuX_2_(*p*-tolyl_2_dtc)]” systems (X = Cl and Br) could be synthesized, and the solution-phase behavior of these systems was identical to their dialkyldithiocarbamate analogues.^27^ Hence, a mixture of “[AuCl_2_(*p*-tolyl_2_dtc)]” (with [AuCl_2_(*p*-tolyl_2_dtc)] as the dominant component) was reacted with MeMgBr, affording the desired [AuMe_2_(*p*-tolyl_2_dtc)] in 90% yield. The ^1^H NMR spectrum of this compound contained a single product with Au–Me peaks at ∼1 ppm, satisfactory elemental analysis was obtained, and X-ray quality crystals were grown from warm CH_2_Cl_2_ (Figure 5).

**Figure 5:**
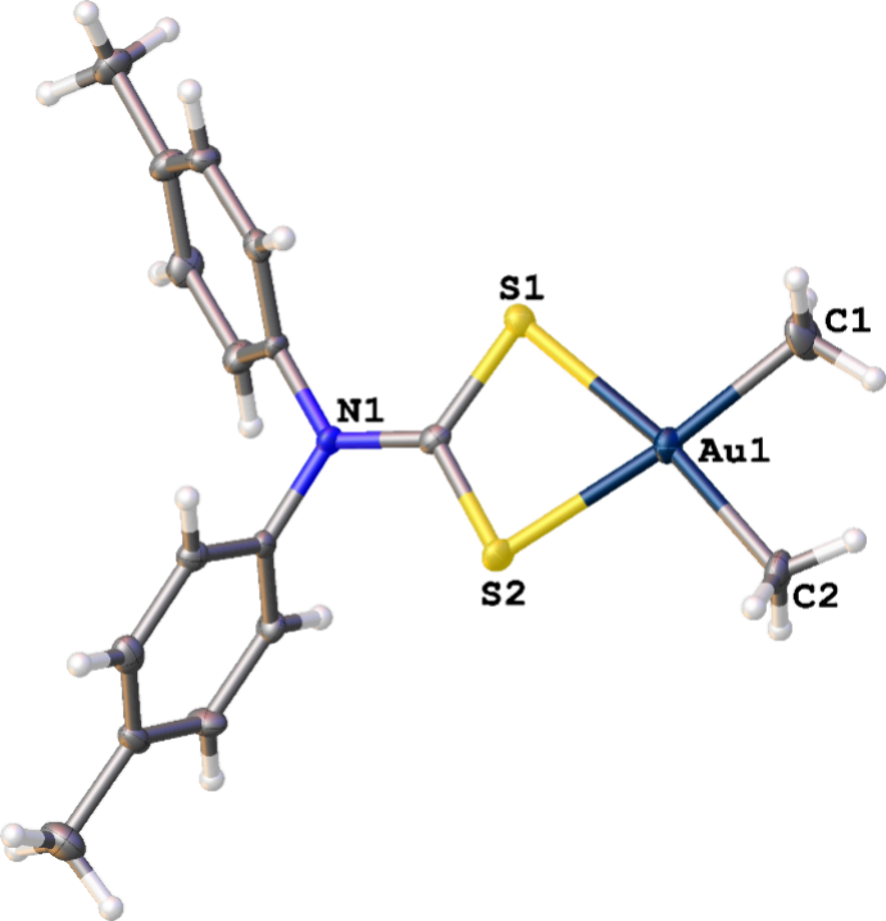
Solid-state structure of [AuMe_2_(*p*-tolyl_2_dtc)] with thermal ellipsoids at the 50% probability level. Selected bond lengths (Å): Au–S1, 2.416(3); Au–S2, 2.401(2); Au–C1, 2.061(10); Au–C2, 2.053(10).

[AuMe_2_(*p*-tolyl_2_dtc)] crystallized in the monoclinic space group *Cc* as a four-coordinate square planar Au­(III) complex. The Au–C and Au–S bond lengths are within the same ranges as those for other examples of [AuMe_2_(dtc)] complexes, and no aurophilic interactions were observed. Instead, CH···Au distances dominated the intermolecular packing; these were ∼0.1 Å longer than in [AuMe_2_(Me_2_dtc)], but the CH···Au angle in [AuMe_2_(*p*-tolyl_2_dtc)] of 171.43° is identical (within experimental error) to that found in [AuMe_2_(Me_2_dtc)]. Due to the strong directionality to these interactions, [AuMe_2_(dtc)] complexes are a promising class of compounds for further investigations into CH···Au­(III) hydrogen bonding.

In addition to the [AuMe_2_(dtc)] complexes, a few examples of Au­(I) dithiocarbamate compounds with the formulation [Au­(dtc)]_*n*_ were synthesized for comparison purposes. These were assumed to be coordination polymers in the solid state based on the crystal structure of [Au­(Et_2_dtc)]_*n*_ ^40^ but may break up into more volatile dimers under heating. A simple salt metathesis reaction between Na­(dtc) and [AuCl­(tht)] (tht = tetrahydrothiophene) furnished the desired Au­(I) compounds in good yield.

## Results and Discussion: Thermogravimetric Analysis

To assess the thermal stability and volatility of the complexes synthesized above, TGA experiments were carried out under the same conditions as those reported by Mäkelä et al. (a flowing N_2_ atmosphere and a 10 °C/min ramp rate). Initially, [AuMe_2_(Et_2_dtc)] was tested to provide a benchmark against the literature data (Figure 6). The observed melting point was 46–50 °C, and a single-step mass loss with onset at ∼120 °C led to a residual mass of ∼20%, indicating that most of the compound was volatilized into the gas phase. While the residual mass was slightly higher, our TGA profile was otherwise consistent with literature data.^8,17^

**Figure 6:**
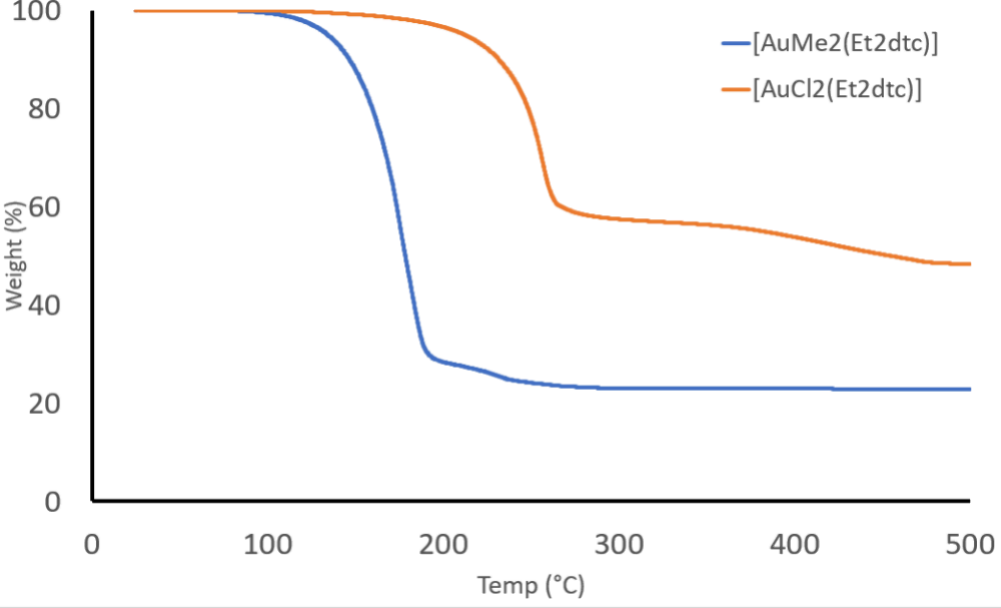
TGA traces of [AuX_2_(Et_2_dtc)] where X = Cl (orange) and X = Me (blue). Conditions: 30–500 °C @ 10 °C/min, N_2_ atmosphere.

It was noteworthy that Mäkelä et al. did not purify their sample of [AuCl_2_(Et_2_dtc)] in their original screening study, which meant that the compound they analyzed was likely a mixture of [AuCl_2_(Et_2_dtc) and [Au­(Et_2_dtc)_2_]­[AuCl_4_], with the latter as the majority component.^8,27^ We reanalyzed this compound using a pure, crystalline sample, but our TGA trace (Figure 6) was very similar to that reported by Mäkelä et al.: a residual mass of ∼50% indicated that this compound likely underwent decomposition to metallic gold since [AuCl_2_(Et_2_dtc)] is 47.3% Au by mass. This indicates that the general class of compounds [AuCl_2_(dtc)] are unsuitable ALD precursors even when used in a pure, crystalline form.

TGA of the [AuMe_2_(dtc)] complexes synthesized in this study (Figure 7) showed that many of these compounds exhibited a single-step mass loss. There was also an increase in residual mass, which roughly correlated to the increasing molecular mass of the compounds. The *p*-tolyl_2_dtc derivative had a clear two-step mass loss, which indicated a more complex decomposition profile rather than just volatilization of the precursor. Given the high molecular weight of diaryldithiocarbamate ligands, coupled with the nontrivial synthesis of these compounds, we concluded that compounds of the type [AuMe_2_(Ar_2_dtc)] (Ar = aryl) were unlikely to make good ALD precursors and were not investigated further.

**Figure 7:**
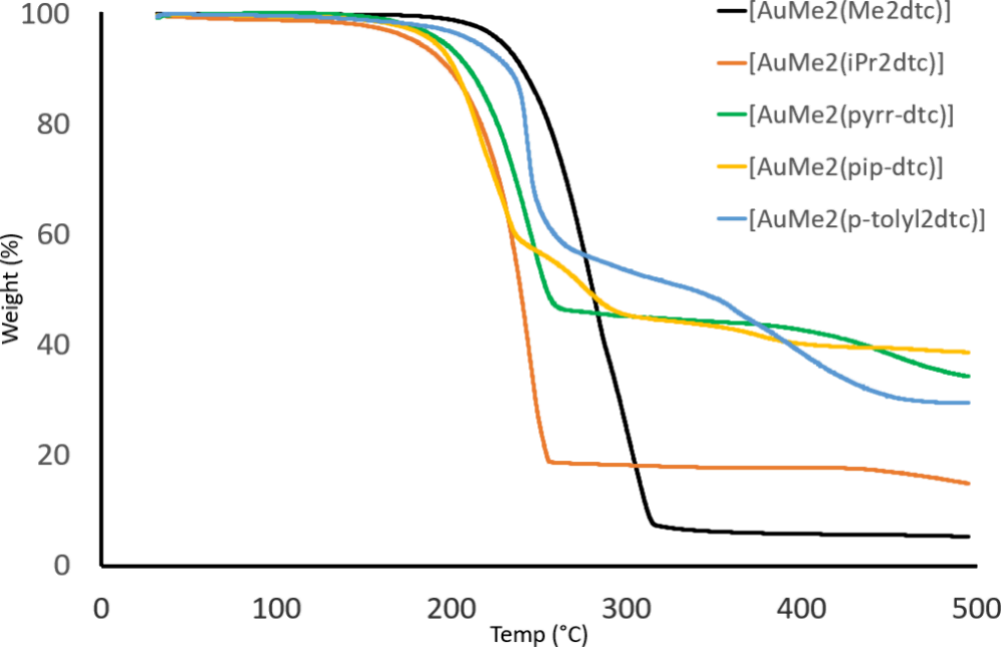
TGA traces of [AuMe_2_(R_2_dtc)] where R = Me (black), R = ^*i*^Pr (orange), R_2_ = pyrrolidine (green), R_2_ = piperidine (yellow), and R = *p*-tolyl (blue). Conditions: 30–500 °C @ 10 °C/min, N_2_ atmosphere.

The lowest residual mass of ∼5% was observed for [AuMe_2_(Me_2_dtc)], indicating that this compound has comparable volatility with [AuMe_2_(Et_2_dtc)].^8,17^ Given that both Na­(Et_2_dtc) and Na­(Me_2_dtc) are commercially available starting materials and the synthetic route to [AuMe_2_(dtc)] for both compounds is identical, [AuMe_2_(Me_2_dtc)] could also be a viable precursor to the ALD of gold thin films. However, it should be noted that the purification of [AuCl_2_(Me_2_dtc)] is problematic due to poor solubility;^27^ hence, the overall yield of [AuMe_2_(Et_2_dtc)] starting from “AuCl_3_” is higher than [AuMe_2_(Me_2_dtc)].

The remaining compounds [AuMe_2_(^*i*^Pr_2_dtc)], [AuMe_2_(pyrr-dtc)], and [AuMe_2_(pip-dtc)] all exhibited a one-step mass loss but with residual masses higher than those of the Me_2_dtc derivative. There seems to be no justification for considering any of these compounds as a promising ALD precursor to gold thin films.

Under the same TGA conditions, all the [Au­(dtc)]_*n*_ precursors that were studied exhibited similar behavior to each other, namely, a single-step mass loss to a residual mass of ∼65% (Figure 8). This is consistent with decomposition to metallic gold rather than volatilization of the compound; for example, [Au­(Et_2_dtc)]_*n*_ contains 57% Au by mass. These results indicate that compounds with the general formula [Au­(dtc)]_*n*_ are not good precursors for the ALD of gold thin films.

**Figure 8:**
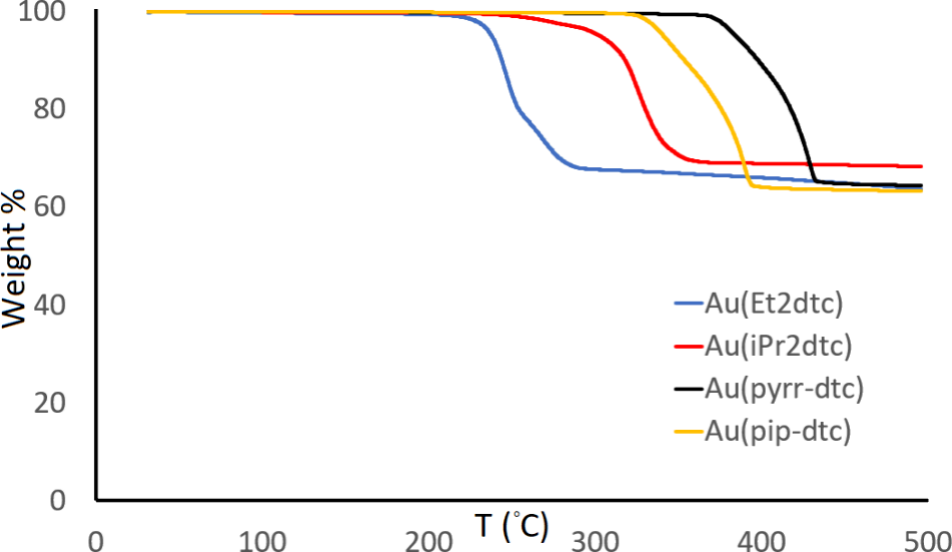
TGA traces of [Au­(R_2_dtc)] where R = Et (blue), R = ^*i*^Pr (red), R_2_ = pyrrolidine (black), and R_2_ = piperidine (yellow). Conditions: 30–500 °C @ 10 °C/min, N_2_ atmosphere.

## Conclusions

A new synthetic route has been developed for the synthesis of a series of compounds [AuMe_2_(dtc)] (dtc = dithiocarbamate) containing a variety of dialkyl-, cyclic alkyl-, and diaryldithiocarbamate ligands. This route is significantly higher-yielding, hence cheaper and less wasteful, than the existing literature route. It is also quicker than existing routes since it is possible to make analytically pure [AuMe_2_(dtc)] in less than 24 h, starting from commercially available “AuCl_3_” and a source of dithiocarbamate. Some of these compounds, notably [AuMe_2_(Me_2_dtc)] and [AuMe_2_(*p*-tolyl_2_dtc)], exhibit interesting structural features in the solid state such as CH···Au­(III) hydrogen bonding with strong directionality.

These compounds were screened by thermogravimetric analysis to determine their suitability as precursors for the atomic layer deposition of gold thin films. Compared to the best literature precursor [AuMe_2_(Et_2_dtc)], analogues with heavier dtc ligands were less volatile or harder to synthesize, so they are unlikely to be good precursors. However, the methyl analogue [AuMe_2_(Me_2_dtc)] is lighter and has comparable volatility; hence, it should be considered as a promising precursor for the ALD of gold thin films. Conversely, gold­(I) compounds of the type [Au­(dtc)]_*n*_ all decomposed to metallic gold under TGA conditions and are not likely to be good molecular precursors for the ALD of gold thin films.

## Experimental Section

### General Procedure

A crude “[AuCl_2_(dtc)]” mixture (i.e., after a 16 h reflux in acetone to convert the mixture such that the majority was [AuCl_2_(dtc)])^27^ was weighed into a Schlenk flask. For molarity calculations, it was assumed that the solid was entirely composed of [AuCl_2_(dtc)]. The flask was placed under an inert atmosphere, and then dry CH_2_Cl_2_ was added to create a solution of ∼0.02–0.1 M concentration. This was cooled to −78 °C, and MeMgBr (2.0 equiv, 3.0 M solution in Et_2_O) was added dropwise over ∼15 min. The solution was stirred for 10 min at −78 °C before warming to room temperature. After a further 10 min of stirring at room temperature, the reaction was quenched by the addition of EtOH (dropwise until no further gas evolution was observed) and then a 1:1 mixture of H_2_O and EtOH was added (approximately the same volume as the initial volume of CH_2_Cl_2_). Following a standard aqueous workup, the combined organic layers were dried (MgSO_4_), filtered, and volatiles removed *in vacuo*. The solid residue was then dissolved in a minimum amount of EtOH and passed through a short silica plug (eluent 1:1 CH_2_Cl_2_:EtOH) where a yellow band containing the product eluted within 2 column volumes (*R*_f_ = 0.5). The yellow fractions were combined and filtered, and volatiles were removed. For compounds in which an orange-yellow oil resulted, trituration with *n*-hexane removed the residual EtOH to give a pale-yellow solid powder. The products can be recrystallized from gently warmed CH_2_Cl_2_.

### [AuMe_2_(Me_2_dtc)]

The general procedure was followed, using “[AuCl_2_(Me_2_dtc)]” (150 mg, 0.39 mmol) and MeMgBr (0.26 mL of a 3.0 M solution in Et_2_O, 0.78 mol, 2 equiv). Yield: 107 mg (79%). X-ray quality crystals were grown by slowly cooling a warm, concentrated CH_2_Cl_2_ solution. ^1^H and ^13^C­{^1^H} NMR spectra were consistent with literature data.^16^

^1^H NMR (400.1 MHz, CDCl_3_): δ 3.31 (s, 6H, N–CH_3_), 1.03 (s, 6H, Au–CH_3_).

^13^C­{^1^H} NMR (100.6 MHz, CDCl_3_): δ 206.98 (N*C*S_2_), 40.44 (N–CH_3_), 5.61 (Au–CH_3_).

Elemental analysis calc. for C_5_H_12_AuNS_2_ (347.23): C, 17.29%; H, 3.48%; N, 4.03%. Found: C, 17.60%; H, 3.42%; N, 4.16%.

### [AuMe_2_(Et_2_dtc)]

The general procedure was followed, using “[AuCl_2_(Et_2_dtc)]” (70 mg, 0.16 mmol) and MeMgBr (0.11 mL of a 3.0 M solution in Et_2_O, 0.34 mmol, 2 equiv). Yield: 56 mg (88%). ^1^H NMR spectroscopic data agree with literature data in C_6_D_6_, and for completeness, the data are also reported here in CDCl_3_.^8^

^1^H NMR (400.1 MHz, CDCl_3_): δ 3.70 (q, ^3^ *J*_HH_ = 7.2 Hz, 4H, CH_2_), 1.33 (t, ^3^ *J*_HH_ = 7.2 Hz, 6H, CH_3_), 1.03 (s, 6H, Au–CH_3_).

### [AuMe_2_(^*i*^Pr_2_dtc)]

The general procedure was followed, using “[AuCl_2_(^*i*^Pr_2_dtc)]” (120 mg, 0.27 mmol, 1 equiv) and MeMgBr (0.18 mL, 3.0 M solution in Et_2_O, 0.27 mmol, 2 equiv). This reaction was conducted in an ampule fitted with a J. Young tap to avoid silicon grease contamination. Yield: 81 mg (74%).

^1^H NMR (400.1 MHz, CDCl_3_): δ 5.5–3.77 (v br s, 2H, CH), 1.49 (v br, 12 H, CH_3_), 0.98 (s, 6H, Au–CH_3_).

^13^C­{^1^H} NMR (100.6 MHz, CDCl_3_): δ 204.71 (NCS_2_), 52.05 (NCH), 19.85 (CH*C*H_3_), 5.92 (Au–CH_3_).

Elemental analysis calc. for C_9_H_20_AuNS_2_ (403.33): C, 26.80%; H, 5.00%; N, 3.47%. Found: C, 26.52%; H, 4.81%; N, 3.59%.

### [AuMe_2_(pyrr-dtc)]

The general procedure was followed, using “[AuCl_2_(pyrr-dtc)]” (120 mg, 0.29 mmol, 1 equiv) and MeMgBr (0.19 mL of a 3.0 M solution in Et_2_O, 0.58 mmol, 2 equiv). Yield: 84 mg (91%). X-ray quality crystals were grown by slowly cooling a warm, concentrated CH_2_Cl_2_ solution.

^1^H NMR (400.1 MHz, CDCl_3_): δ 3.81–3.75 (m, 4H, N–CH_2_), 2.10–2.04 (m, 4H, CH_2_), 1.03 (s, 6H, Au–CH_3_).

^13^C­{^1^H} NMR (100.6 MHz, CDCl_3_): δ 201.88 (NCS_2_), 50.58 (N–CH_2_), 24.73 (CH_2_), 6.29 (Au–CH_3_).

Elemental analysis calc. for C_7_H_14_AuNS_2_ (373.27): C, 22.52%; H, 3.78%; N, 3.75%. Found: C, 22.42%; H, 3.73%; N, 4.07%.

### [AuMe_2_(pip-dtc)]

The general procedure was followed, using “[AuCl_2_(pip-dtc)]” (37 mg, 0.09 mmol, 1 equiv) and MeMgBr (0.06 mL of a 3.0 M solution in Et_2_O, 0.17 mmol, 2 equiv). Yield: 33 mg (98%). X-ray quality crystals were grown by slowly cooling a warm, concentrated CH_2_Cl_2_ solution.

^1^H NMR (400.1 MHz, CDCl_3_): δ 3.86–3.83 (m, 4H, N–CH_2_), 1.81–1.75 (m, 2H, CH_2_), 1.73–1.68 (m, 4H, CH_2_), 1.03 (s, 6H, Au–CH_3_).

^13^C­{^1^H} NMR (100.6 MHz, CDCl_3_): δ 204.09 (NCS_2_), 49.65 (N–CH_2_), 25.62 (CH_2_), 24.57 (CH_2_), 6.11 (Au–CH_3_).

Elemental analysis calc. for C_8_H_16_AuNS_2_ (387.29): C, 24.81%; H, 4.16%; N, 3.62%. Found: C, 25.21%; H, 4.02%; N, 3.61%.

### [AuMe_2_(*p*-tolyl_2_dtc)]

The general procedure was followed, using “[AuCl_2_(*p*-tolyl_2_dtc)]” (58 mg, 0.11 mmol, 1 equiv) and MeMgBr (0.07 mL of a 3.0 M solution in Et_2_O, 0.22 mmol, 2 equiv). Yield: 49 mg (90%).

^1^H NMR (400.1 MHz, CDCl_3_): δ 7.30 (d, 4H, *J* = 6.8 Hz, CH), 7.23 (d, 4H, *J* = 6.8 Hz, CH), 2.36 (s, 6H, Ar–CH_3_), 1.00 (s, 6H, Au–CH_3_).

^13^C­{^1^H} NMR (100.6 MHz, CDCl_3_): δ 139.39 (C), 139.13 (C), 130.44 (CH), 127.09 (CH), 21.35 (Ar–CH_3_), 5.52 (Au–CH_3_). No signal was observed for the NCS_2_ carbon atom.

Elemental analysis calc. for C_17_H_20_AuNS_2_ (499.41): C, 40.88%; H, 4.04%; N, 2.80%. Found: C, 41.09%; H, 3.98%; N, 2.95%.

## Supplementary Material



## Notes

General experimental details, extra crystallographic discussion and information, spectral data, and thermogravimetric analytical data (PDF)
