# Supplementary material for: A Higher-Yielding Route for the Synthesis of Molecular Precursors to Thin Films of Gold by Atomic Layer Deposition
Source: Organometallics. 2025 Sep 16;44(19):2202–8. doi: 10.1021/acs.organomet.5c00242 (PMC12522145; doi:10.1021/acs.organomet.5c00242)
Supplement: Supplementary file 1 [file om5c00242_si_001.pdf]

Supporting Information

for

A Higher-Yielding Route for the Synthesis of Molecular Precursors to Thin Films of Gold by Atomic Layer Deposition

Ryan K. Brown,[a] Joseph N. Bunyan,[a] Ashi Agrawal,[a] Graeme Hogarth,[a] Christopher S. Blackman,[b] Leonardo Santoni,[b] Eva Rimoldi Rudatis,[b] Caroline E. Knapp,[b] and David Pugh*[a]

a: Department of Chemistry, King's College London, Britannia House, 7 Trinity Street, London, SE1 1DB, UK. E-mail David.Pugh@kcl.ac.uk

b: Department of Chemistry, University College London, 20 Gordon Street, London, WC1H 0AJ, UK

Contents

1. General experimental details S2
2. Crystallographic information S4
3. Thermogravimetric analysis data S7
4. NMR spectra S13
5. References S19

1. General Experimental Details

1.1 Materials

Syntheses involving Grignard reagents were conducted under Ar atmosphere using standard Schlenk techniques, apart from [$AuMe_2(^iPr_2dtc)$] which was conducted in a greaseless environment. CH_2Cl_2 was dried using a solvent purification system (SPS) and stored over activated 3 Å molecular sieves under an Ar atmosphere before use.

Na[$AuCl_4$] and H[$AuCl_4$] (Fisher), MeMgBr (3.0 M solution in Et_2O), Na(Me_2dtc)·2(H_2O), Na(Et_2dtc)·3(H_2O) and CS_2 (all Sigma/Merck) were purchased from the companies indicated and used as received. Secondary amines were purchased from common suppliers (typically Sigma/Merck, Fisher, or VWR) and distilled from CaH_2 prior to use unless they were already colorless and pure by ^{1}H NMR spectroscopy. Silica gel (technical grade, 300–400 mesh particle size) for column chromatography and Celite® were both purchased from Fisher.

Na(dtc) and K(dtc) salts were synthesized by a standard literature procedure.[S1] Identities and purity of products were confirmed through comparison to known literature data: Na($^iPr_2$dtc),[S2] Na(pyrr-dtc),[S3] Na(pip-dtc).[S4] Li(p-tolyl$_2$dtc) was synthesized from a literature procedure.[S5] The "[$AuCl_2$(dtc)]" mixtures were synthesized as described in the literature.[S6] For the purposes of molarity calculations, it was assumed that each mixture was composed entirely of the neutral [$AuCl_2$(dtc)] since it was not always possible to accurately measure the proportions of each component (notably for the cyclic dithiocarbamate derivatives). [AuCl(tht)] was synthesised from H[$AuCl_4$] by a literature procedure.[S7] Au(I) dithiocarbamate complexes were synthesized by reacting M(dtc) with one molar equivalent of [AuCl(tht)] in CH_2Cl_2. A coloured suspension formed which was stirred for 24 hours, then isolated by filtration, washed with CH_2Cl_2 (3 x 5 mL) and dried *in vacuo*. Identities and purity of products were confirmed by comparison to literature data: [Au(Et_2dtc)]$_n$,[S8] [Au($^iPr_2$dtc)]$_n$,[S9] [Au(pyrr-dtc)]$_n$,[S8] [Au(pip-dtc)]$_n$.[S10]

1.2 Instrumentation

^{1}H NMR and ^{13}C{^{1}H} NMR spectra were recorded at 298 K on a Bruker Ascend 400 spectrometer operating at 400.1 MHz (^{1}H). Spectra were obtained in $CDCl_3$ (used as received from Merck) and are referenced to the residual protio-solvent signal. The reported values for ^{1}H and ^{13}C{^{1}H} NMR data are as follows: chemical shift (δ, ppm), multiplicity (where s = singlet, d = doublet, t = triplet, m = multiplet), integration (not ^{13}C), and coupling constant (*J*, Hz). 2D correlation spectroscopy (COSY, HSQC, HMBC) were used routinely to aid assignment of the 1D signals but are not documented herein.

Elemental analysis was conducted at London Metropolitan University. Samples were weighed with a Mettler Toledo high-precision scale and analyzed using a Thermo Scientific FLASH 2000. Values reported are averages of duplicate runs.

TGA data were obtained on a Perkin-Elmer STA6000 with sensitivity of 0.1 mg and N_2 as shield gas. Run details: temperature range 25–500 °C at a ramp rate of 10 °C/min under a flowing N_2 atmosphere. All analyses were conducted in ceramic crucibles.

2. Crystallographic Information

General Methods and Instrumentation

We thank the EPSRC UK National Crystallography Service at the University of Southampton for the collection of crystallographic data.[S11]

Source for all [$AuMe_2$(dtc)] complexes: a Rigaku FRE$^+$ diffractometer operating with Mo-K_α radiation, (0.71073 Å).

Source for [Au(^{i}Pr_2dtc)$_2$]Cl·H_2O: an XtaLAB AFC11 (RCD3) quarter-chi single diffractometer operating with Cu-K_α radiation (1.54184 Å).

All structures: diffractometers were equipped with HF Varimax confocal mirrors, HG Saturn 724$^+$ detector, and an Oxford Cryosystems low-temperature device operating at 100(1) K.

Datasets were processed using CrysAlisPro[S12] and solutions were solved and refined using Olex-2.[S13] CCDC reference numbers 2469236 [$AuMe_2$(Me_2dtc)], 2469237 [$AuMe_2$(pyrr-dtc)], 2469238 [$AuMe_2$(pip-dtc)], 2469239 [$AuMe_2$(p-tolyl$_2$dtc)], and 2469240 [Au(^{i}Pr_2dtc)$_2$]Cl·H_2O contain crystallographic data in CIF format, which is summarized in Table S1.

Additional crystallographic experimental details

The NMe_2 groups in [$AuMe_2$(Me_2dtc)] were initially placed using an idealised model (AFIX 137) but were subsequently modelled with no restrictions. During refinement cycles no significant movement of any hydrogen was observed, even H5A (the closest contact to Au), hence the hydrogens were added using an idealised model (AFIX 137) for final refinement.

[$AuMe_2$(pyrr-dtc)] was twinned, with a small minor component (1:9 ratio) present. No other restraints were needed.

No special details were noted for [$AuMe_2$(pip-dtc)], [$AuMe_2$(p-tolyl$_2$dtc)], or [Au(^{i}Pr_2dtc)$_2$]Cl·H_2O.

Table S1: CIF data for the X-ray structures reported in this paper.

	**[AuMe$_2$(Me$_2$dtc)]**	**[AuMe$_2$(pyrr-dtc)]**	**[AuMe$_2$(pip-dtc)]**	**[AuMe$_2$(p-tolyl$_2$dtc)]**	**[Au(^{i}Pr$_2$dtc)$_2$]Cl·H$_2$O**
Empirical formula	$C_5H_{12}AuNS_2$	$C_7H_{14}AuNS_2$	$C_8H_{16}AuNS_2$	$C_{17}H_{20}AuNS_2$	$C_{14}H_{30}AuClN_2OS_4$
Formula weight (Å)	347.24	373.28	387.30	499.43	603.05
Crystal system	triclinic	triclinic	monoclinic	monoclinic	monoclinic
Space group	*P*–1	*P*–1	$P2_1/c$	*Cc*	*I*2/*a*
a (Å)	6.5095(3)	6.4224(1)	6.6544(2)	5.4579(2)	13.8775(1)
b (Å)	7.7011(3)	7.8835(2)	21.8122(5)	21.3571(8)	11.8387(1)
c (Å)	10.0911(4)	11.3258(2)	8.2196(2)	15.0367(4)	27.1071(3)
α (°)	78.718(3)	91.186(2)	90	90	90
β (°)	72.852(4)	103.165(2)	106.254(3)	94.038(3)	96.930(1)
γ (°)	66.386(4)	113.280(2)	90	90	90
Volume (Å^3)	441.17(4)	508.961(19)	1145.36(5)	1748.4(1)	4420.93
Z	2	2	4	4	8
Density (calc.) (g/cm^3)	2.614	2.436	2.246	1.897	1.812
Absorption coefficient	17.068	14.804	13.161	8.646	17.182
F(000)	320	348	728	960	2368
2θ range (°)	4.24 to 57.396	7.066 to 57.444	6.372 to 54.968	6.640 to 54.916	13.718 to 140.54
Reflections collected	19701	5072	49496	10080	31469
Independent reflections	2288	5072	2639	3607	4146
Data / restraints / parameters	2288/0/86	5075/0/104	2639/0/111	3607/2/194	4146/0/219
Goodness-of-fit on F^2	1.057	1.057	1.200	1.109	1.074
Final *R* indices [I>2σ(I)]	R_1 = 0.0213	R_1 = 0.0426	R_1 = 0.0171	R_1 = 0.0292	R_1 = 0.0243
	wR_2 = 0.0569	wR_2 = 0.1247	wR_2 = 0.0348	wR_2 = 0.0746	wR_2 = 0.0649
R indices (all data)	R_1 = 0.0230	R_1 = 0.0451	R_1 = 0.0183	R_1 = 0.0303	R_1 = 0.0246
	wR_2 = 0.0575	wR_2 = 0.1269	wR_2 = 0.0351	wR_2 = 0.0752	wR_2 = 0.0652
Largest diff. peak/hole (eÅ^{-3})	2.35/–2.44	2.19/–2.98	0.99/–1.16	0.94/–1.51	1.71/–1.30

Structure of [Au(^{i}Pr$_2$dtc)$_2$]Cl·H_2O

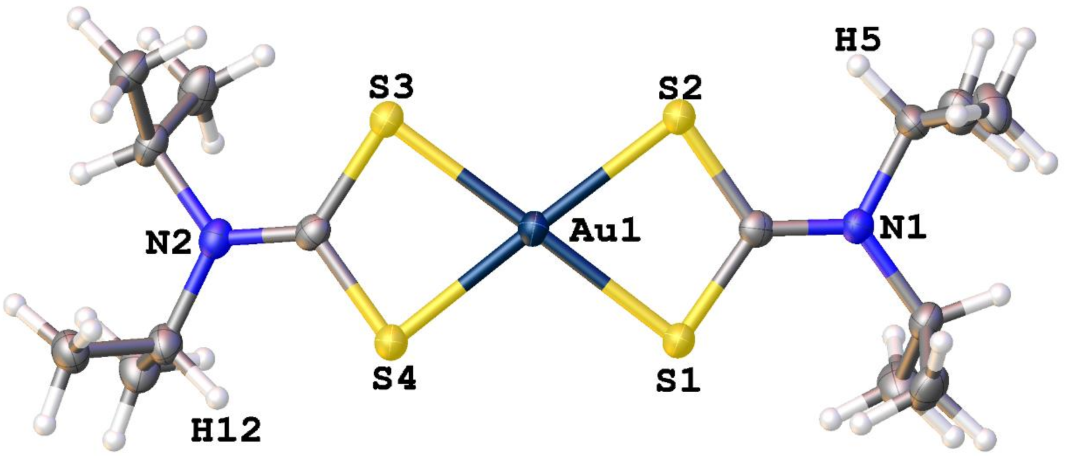

Figure S1: Solid state structure of [Au(^{i}Pr$_2$dtc)$_2$]Cl·H_2O showing the [Au(^{i}Pr$_2$dtc)$_2$]$^+$ cation, with thermal ellipsoids at the 50% probability level. Selected bond lengths (Å): Au–S1 2.3373(8); Au–S2 2.3373(8); Au–S3 2.3352(8); Au–S4 2.3229(8).

[Au(^{i}Pr$_2$dtc)$_2$]Cl·H_2O crystallized in the monoclinic space group *I*2/*a* as a 4-coordinate slightly distorted square planar Au(III) complex (Figure S1). Au–S bond lengths were consistent with those found in the structure of other [Au(dtc)$_2$]$^+$ cations (dtc = Me$_2$dtc, Et$_2$dtc, pyrr-dtc, etc.).[S6] The extended structure shows no evidence of aurophilic interactions (shortest Au···Au distance = 4.35 Å); instead intermolecular S···Au interactions between neighbouring cations are present. The S···Au distance of 3.709 Å is ~0.5 Å less than the sum of van der Waals radii for Au (2.32 Å)and S (1.89 Å).[S14] The chloride ion and lattice water molecule form a hydrogen-bonded ring where two water molecules each act as a double hydrogen-bond donor to two chloride ions (Figure S2). The Cl···H distances of 2.284 Å and 2.251 Å are consistent with other examples of this structural motif.[S15]

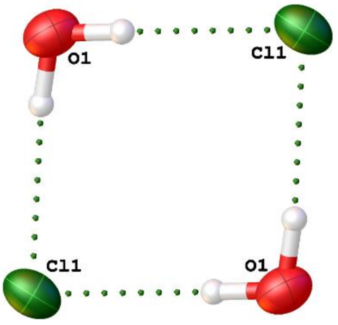

Figure S2: Solid state structure of the hydrogen-bonded chloride anions and lattice water molecule in [Au(^{i}Pr$_2$dtc)$_2$]Cl·H_2O, with thermal ellipsoids at the 50% probability level.

3. Thermogravimetric Analysis Data

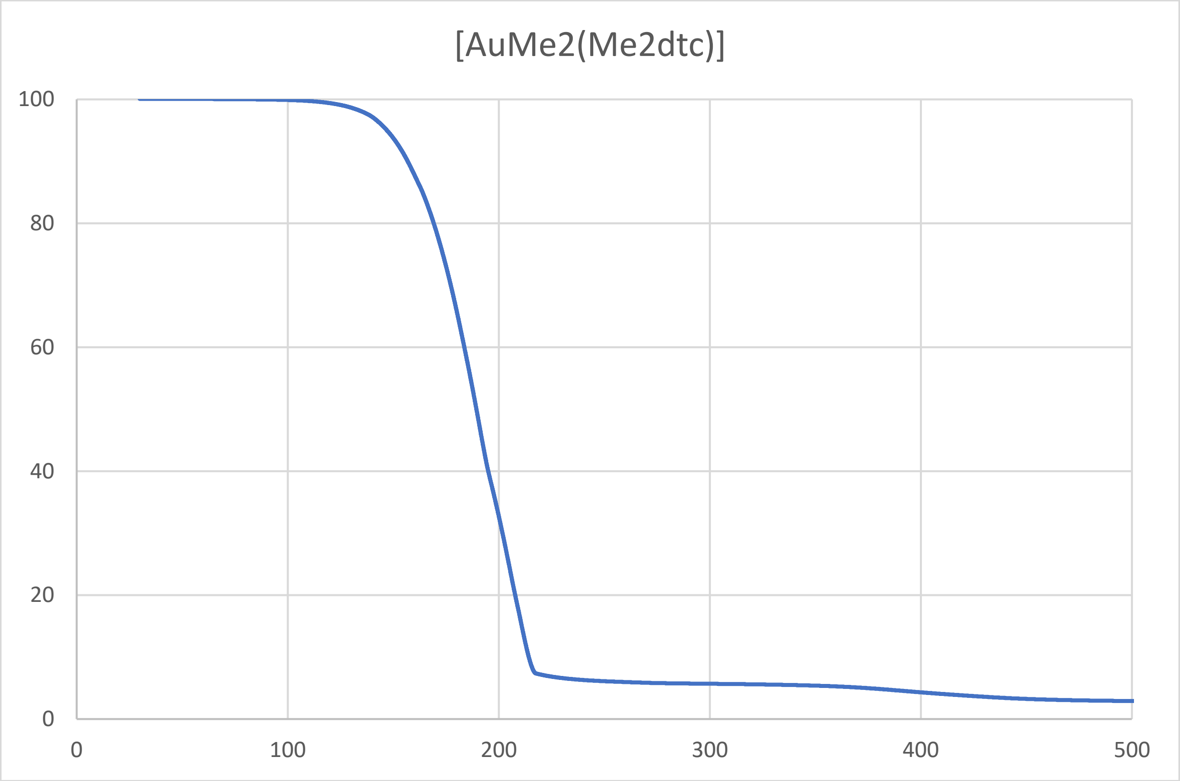

Figure S3: TGA trace for [$AuMe_2(Me_2dtc)$]

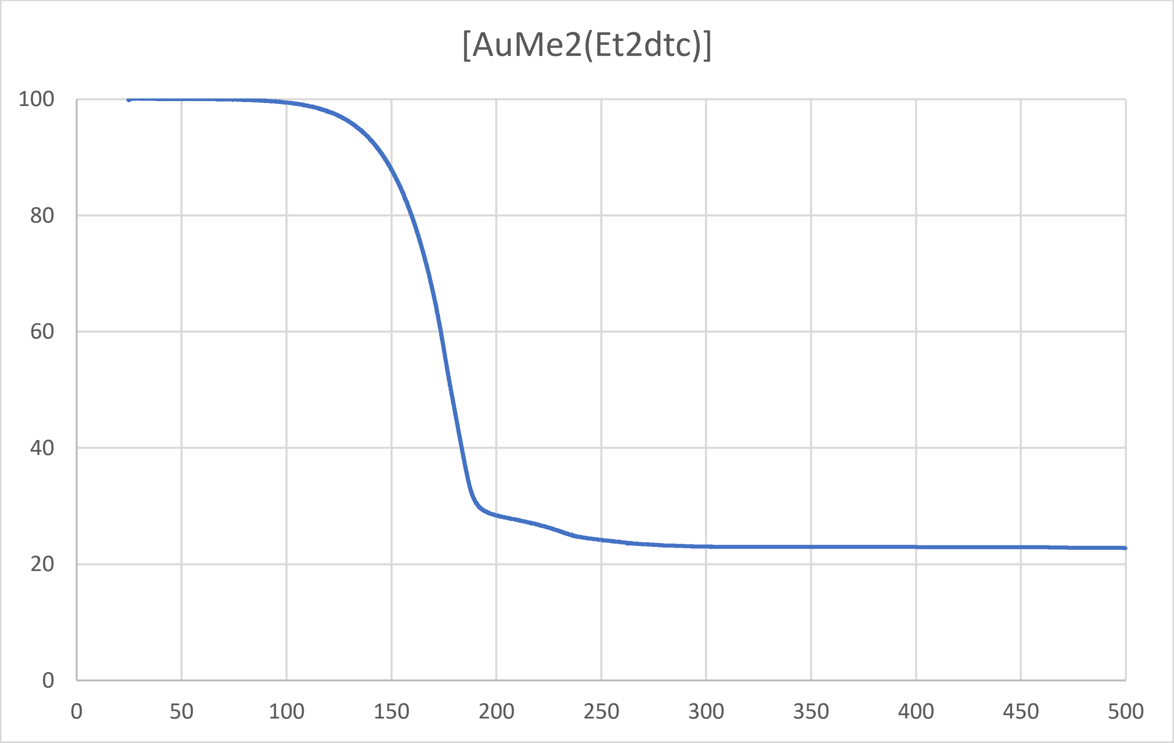

Figure S4: TGA trace for [$AuMe_2(Et_2dtc)$]

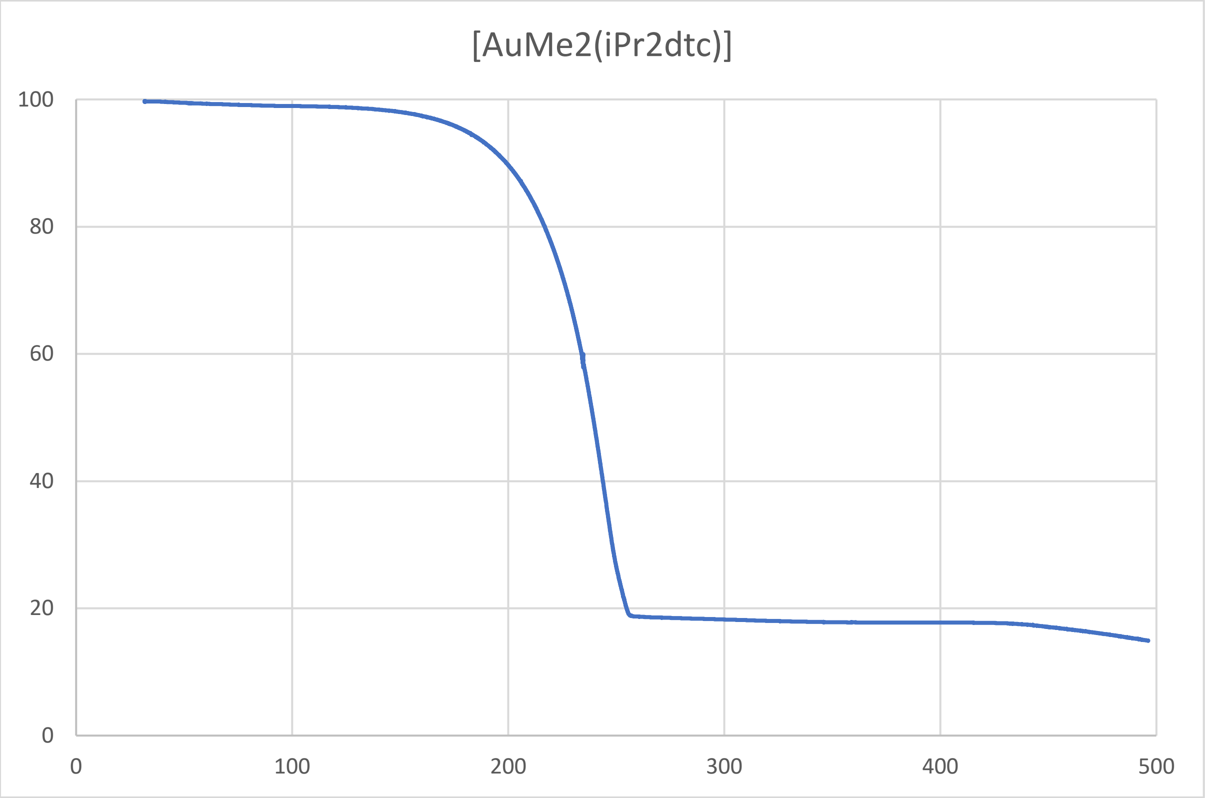

Figure S5: TGA trace for [AuMe$_2$(^{i}Pr$_2$dtc)]

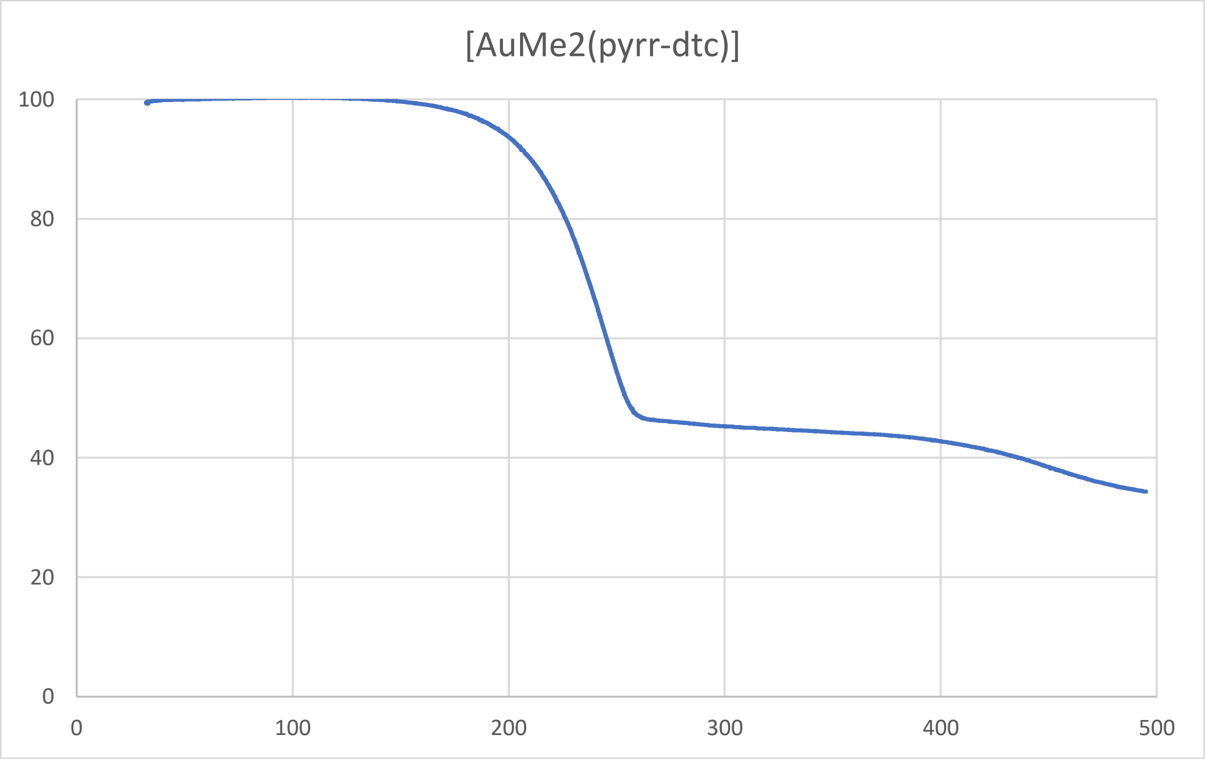

Figure S6: TGA trace for [AuMe$_2$(pyrr-dtc)]

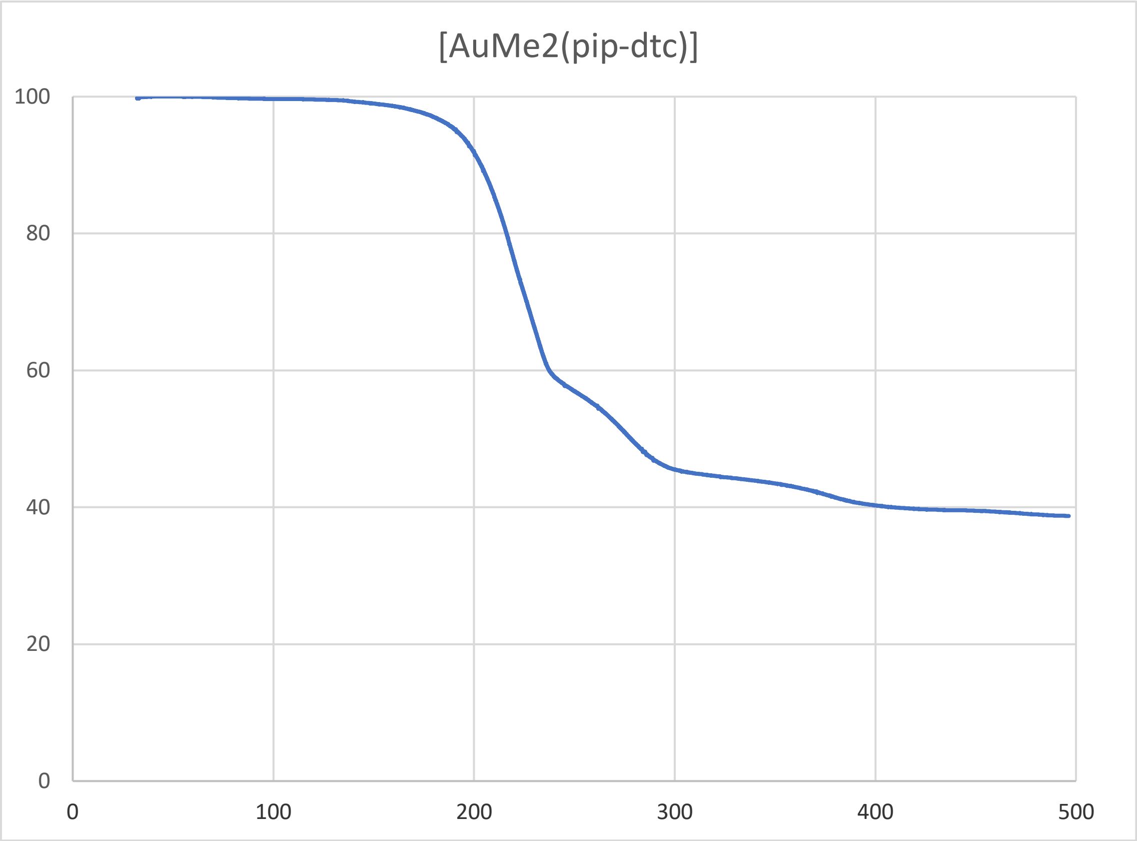

Figure S7: TGA trace for [AuMe$_2$(pip-dtc)]

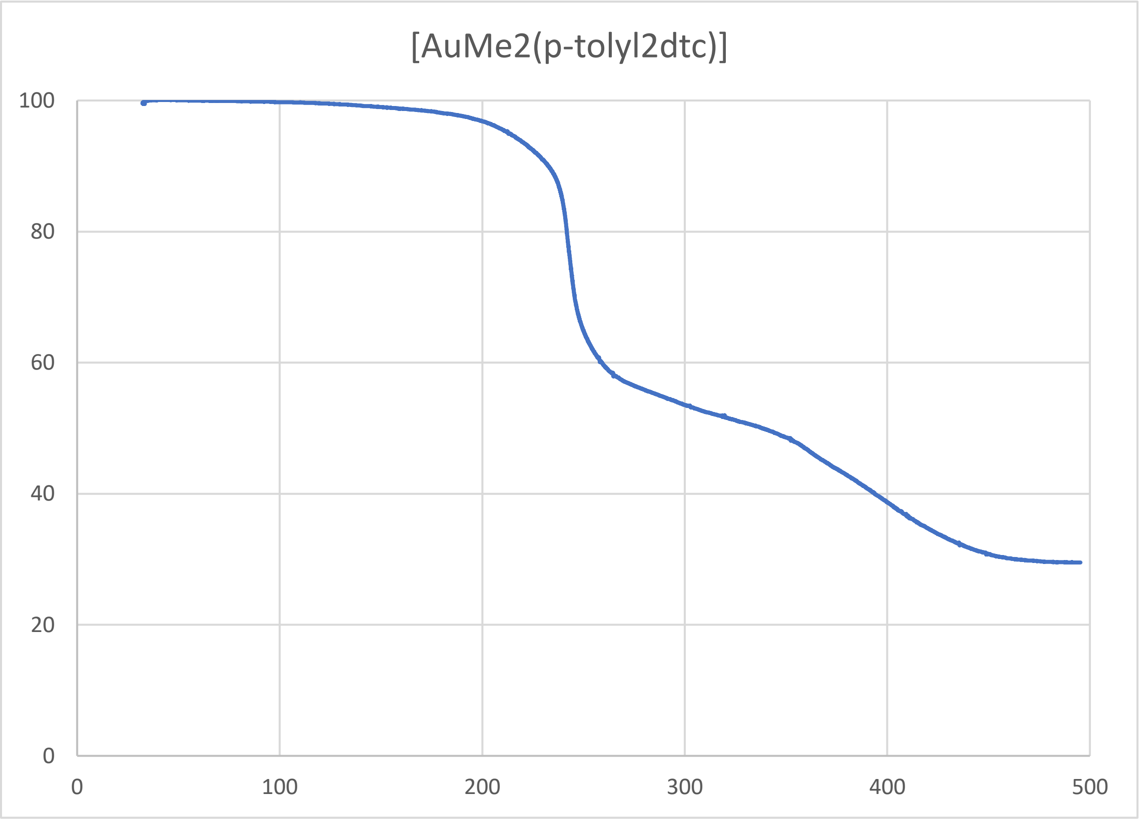

Figure S8: TGA trace for [AuMe$_2$(p-tolyl$_2$dtc)]

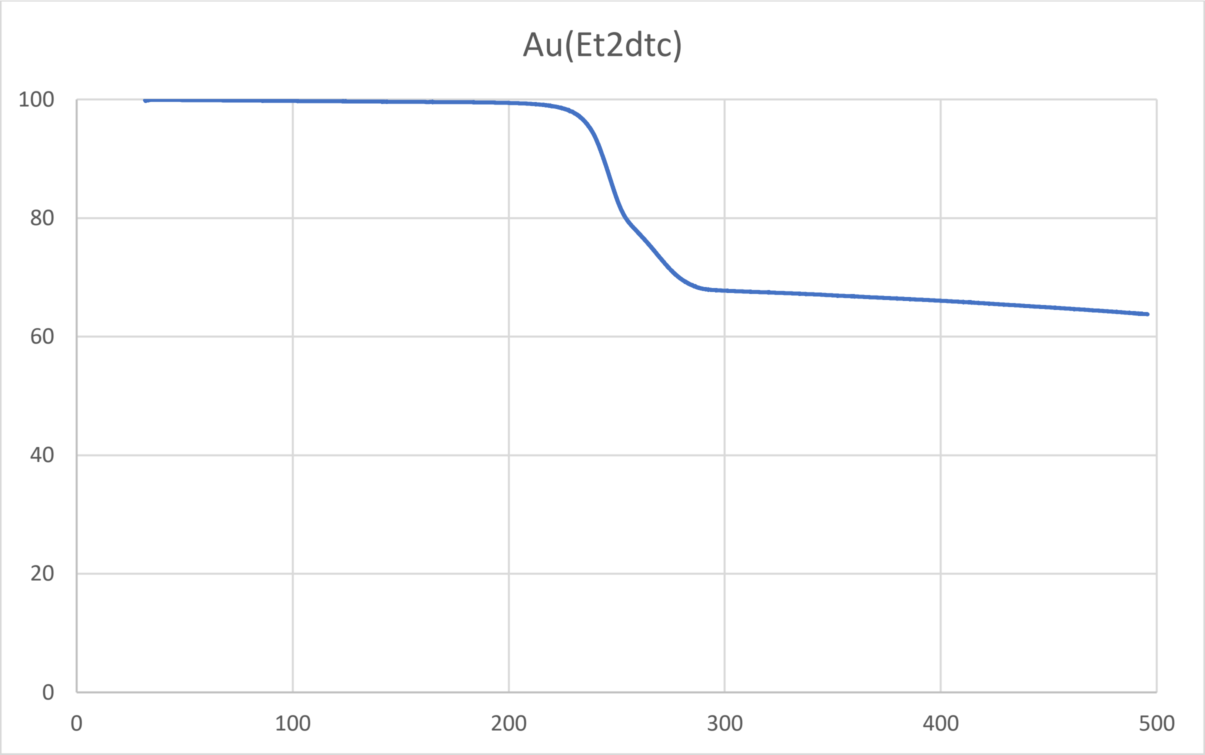

Figure S9: TGA trace for [Au(Et_2dtc)]$_n$

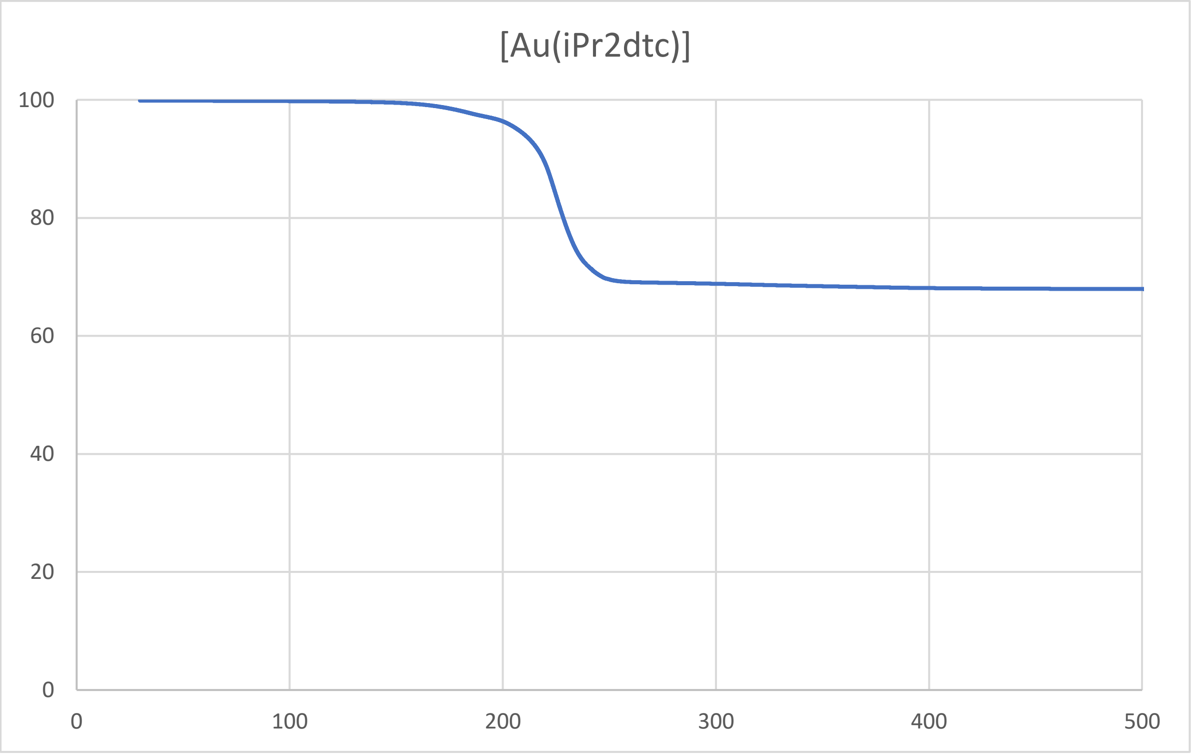

Figure S10: TGA trace for [Au(iPr_2dtc)]$_n$

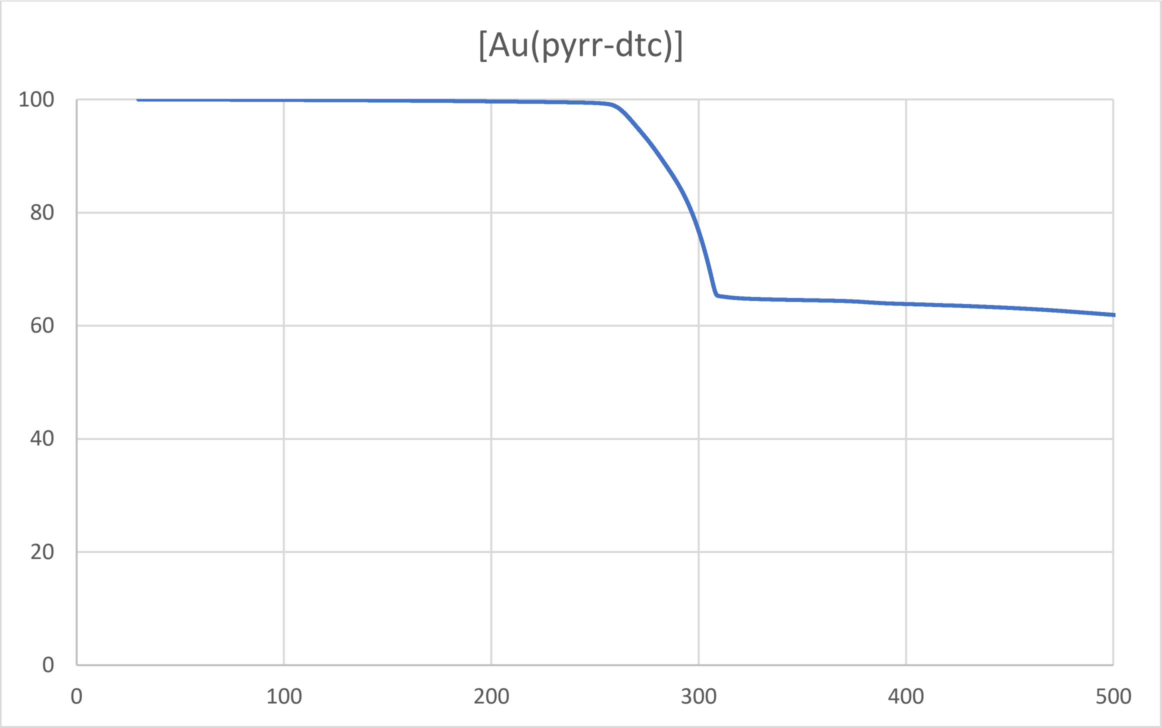

Figure S11: TGA trace for $[Au(pyrr\text{-}dtc)]_n$

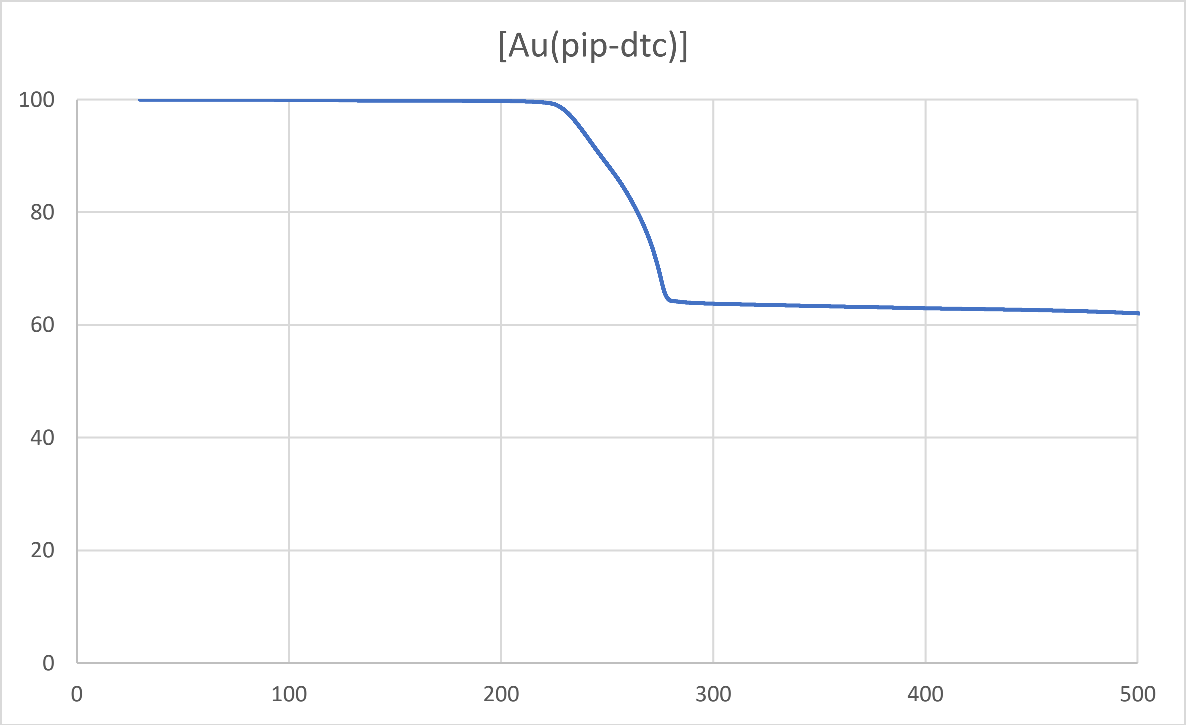

Figure S12: TGA trace for $[Au(pip\text{-}dtc)]_n$

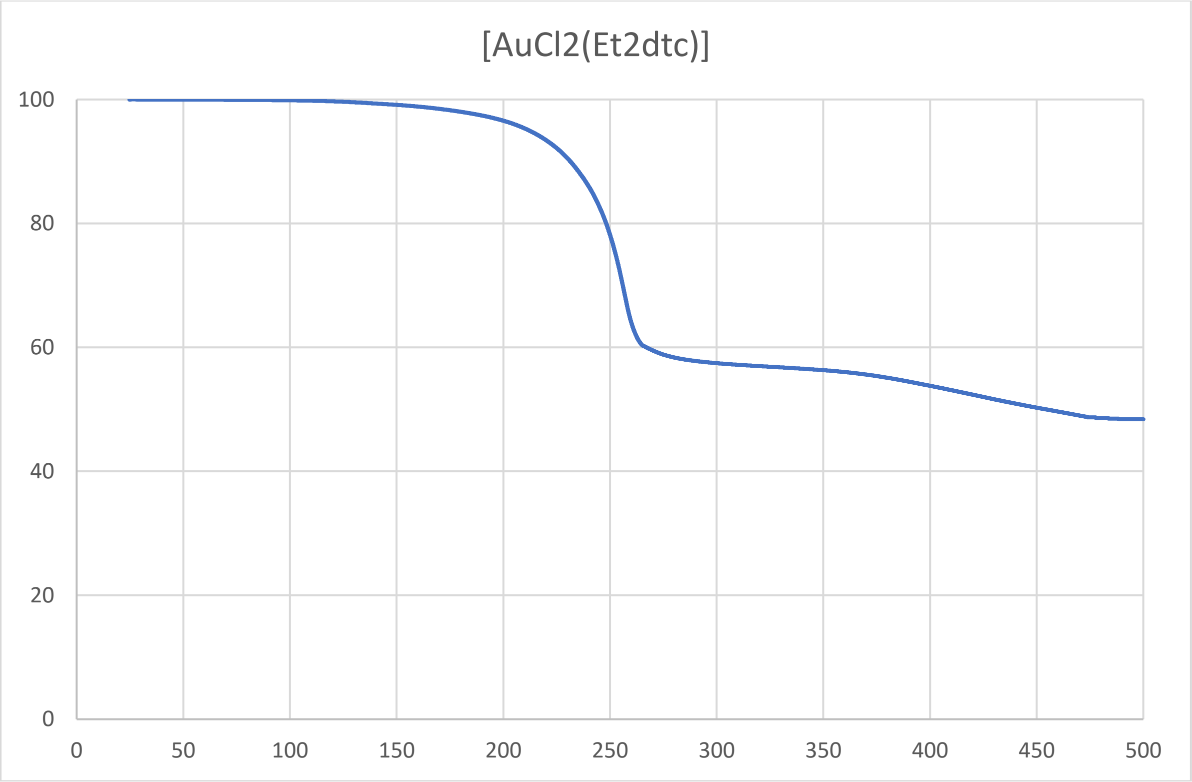

Figure S13: TGA trace for [$AuCl_2(Et_2dtc)$]

4. NMR Spectra

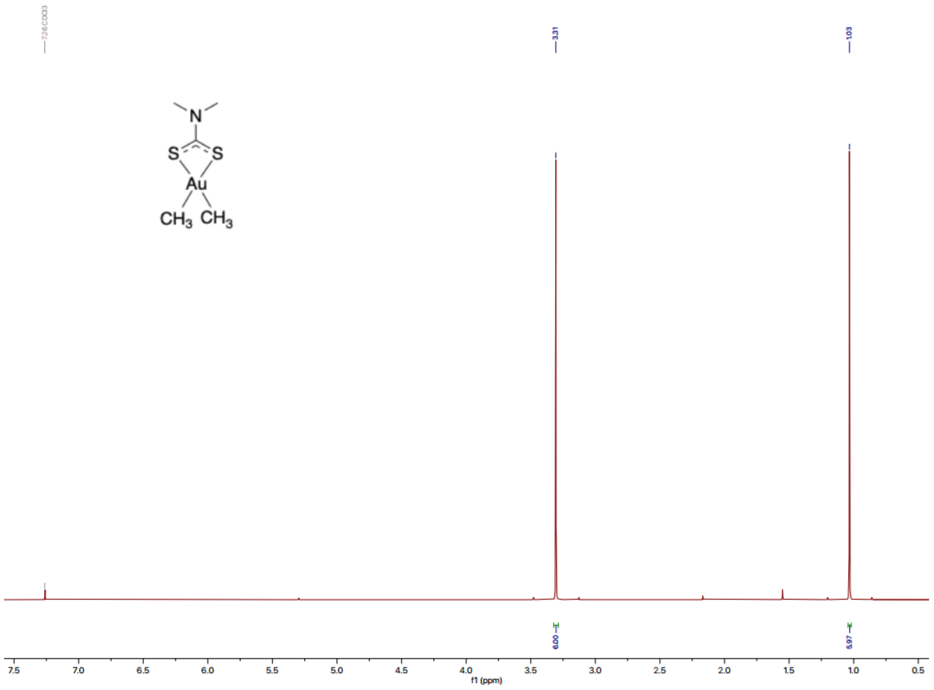

Figure S14: ^{1}H NMR spectrum of $[AuMe_2(Me_2dtc)]$ in $CDCl_3$.

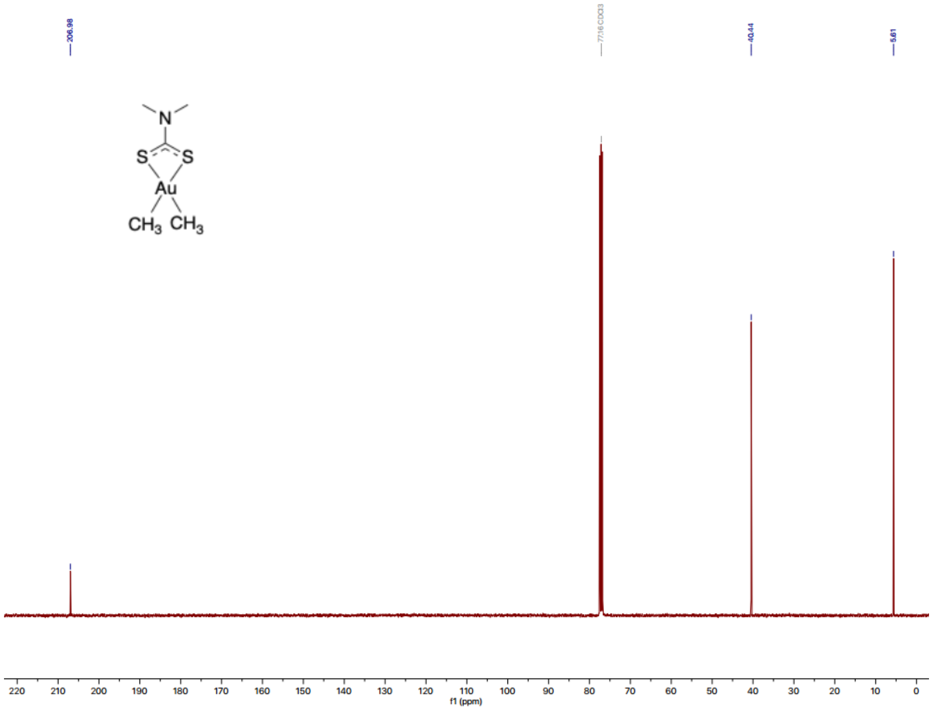

Figure S15: $^{13}C\{^{1}H\}$ NMR spectrum of $[AuMe_2(Me_2dtc)]$ in $CDCl_3$.

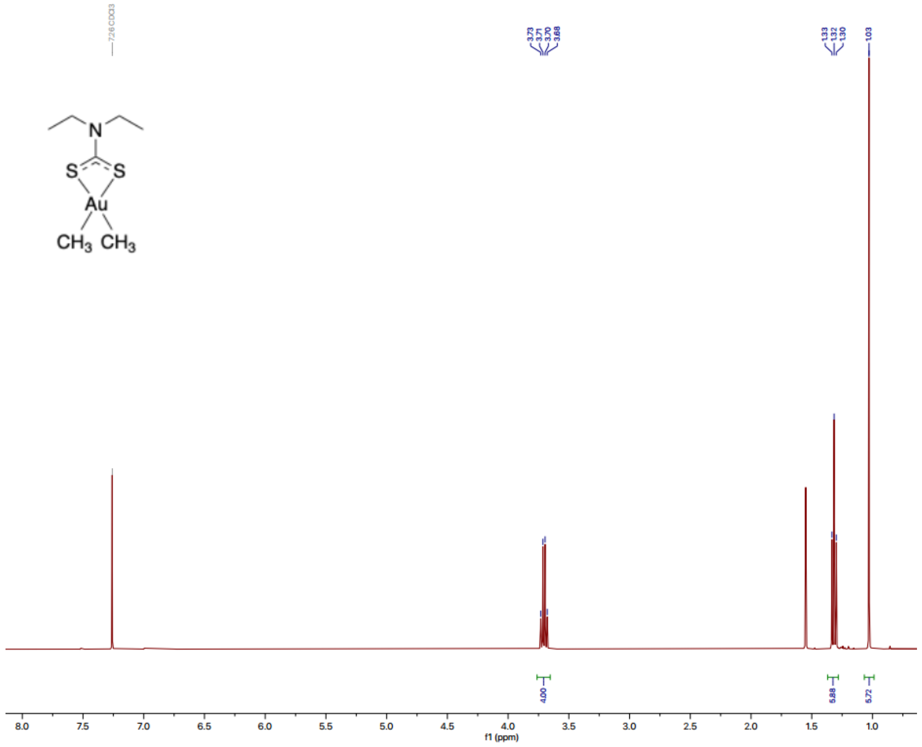

Figure S16: ^{1}H NMR spectrum of [AuMe$_2$(Et$_2$dtc)] in CDCl$_3$.

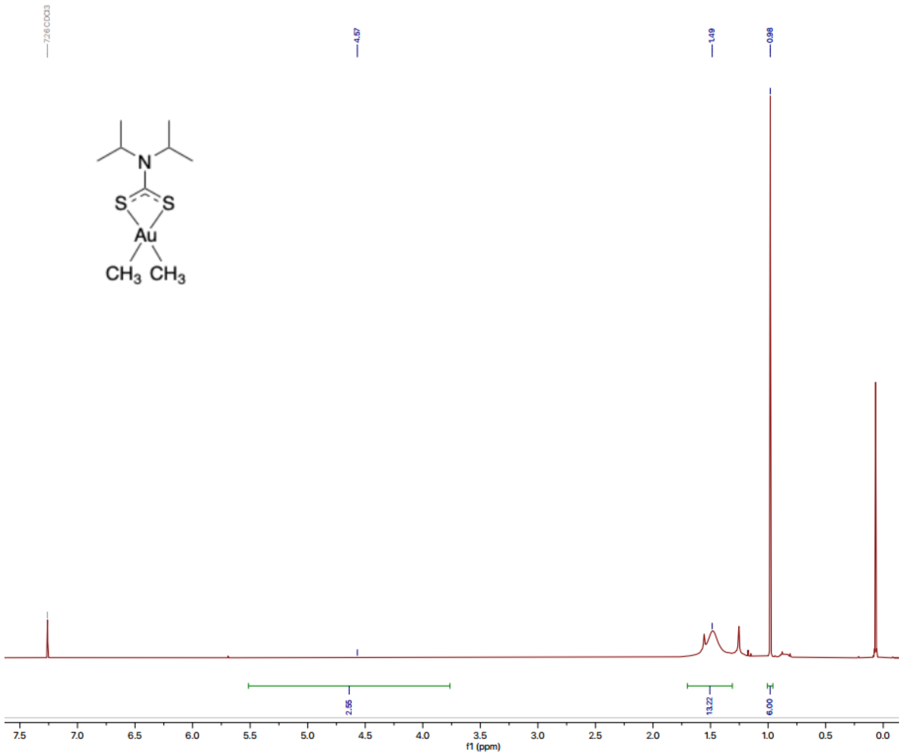

Figure S17: ^{1}H NMR spectrum of [AuMe$_2$(^{i}Pr$_2$dtc)] in CDCl$_3$.

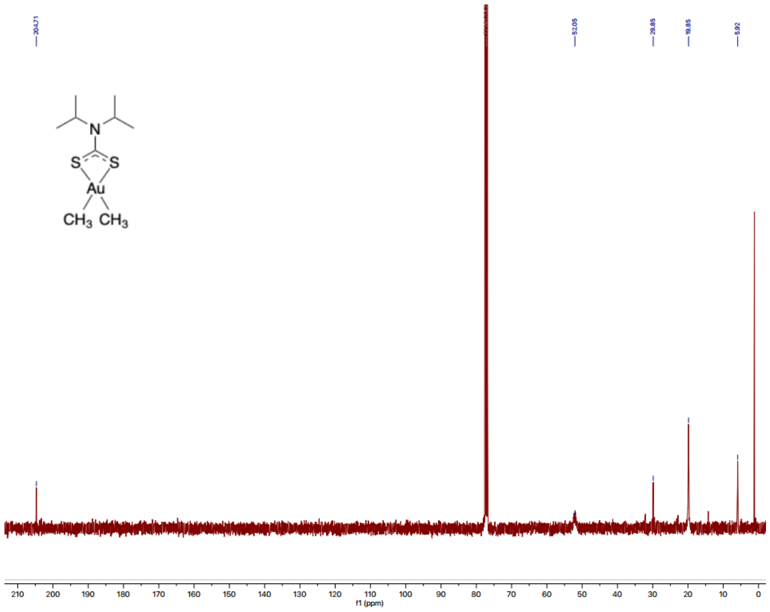

Figure S18: $^{13}C\{^{1}H\}$ NMR spectrum of [$AuMe_2$($^{i}Pr_2$dtc)] in $CDCl_3$.

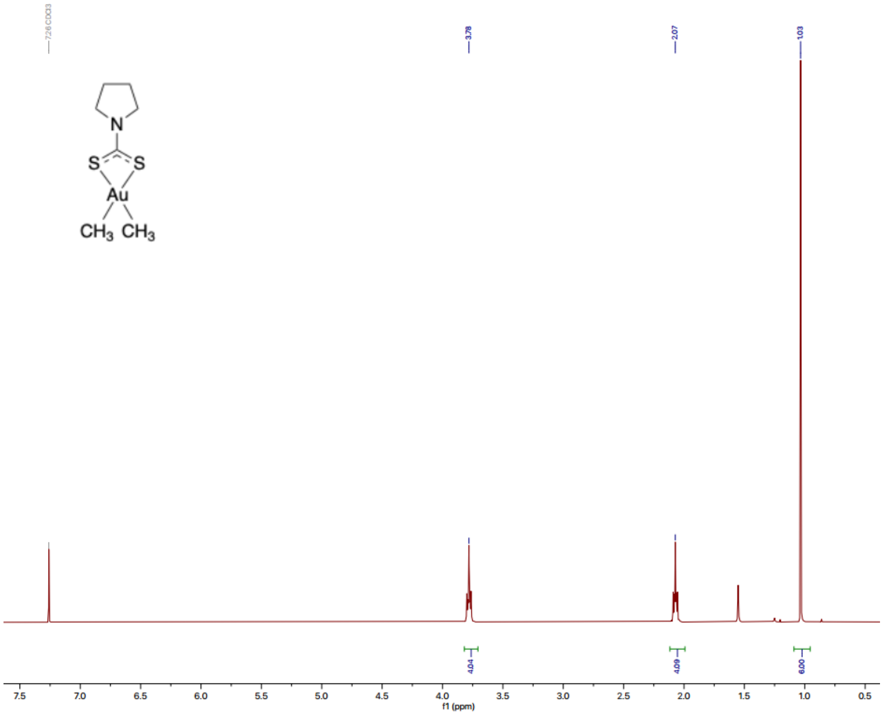

Figure S19: ^{1}H NMR spectrum of [$AuMe_2$(pyrr-dtc)] in $CDCl_3$.

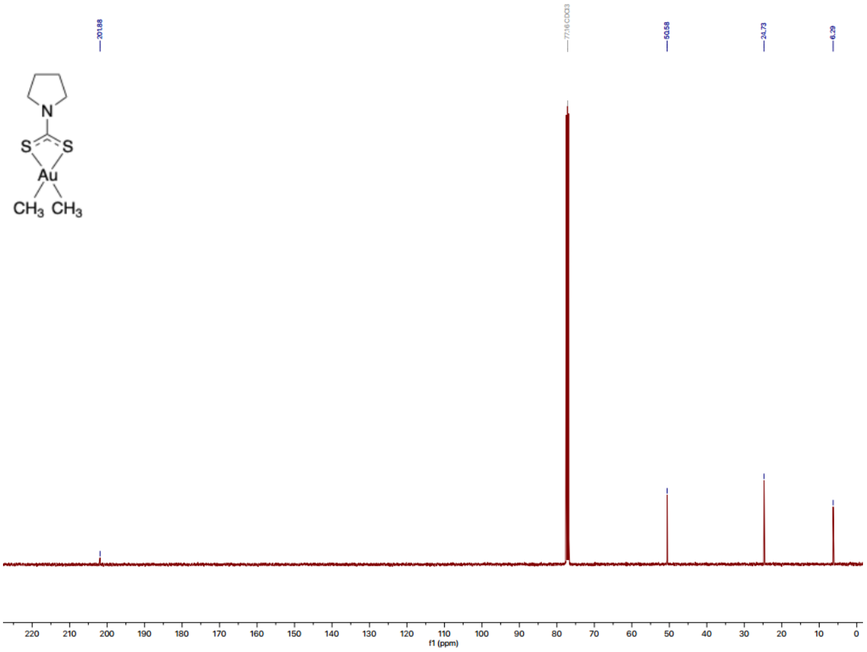

Figure S20: $^{13}C\{^{1}H\}$ NMR spectrum of $[AuMe_2(pyrr\text{-}dtc)]$ in $CDCl_3$.

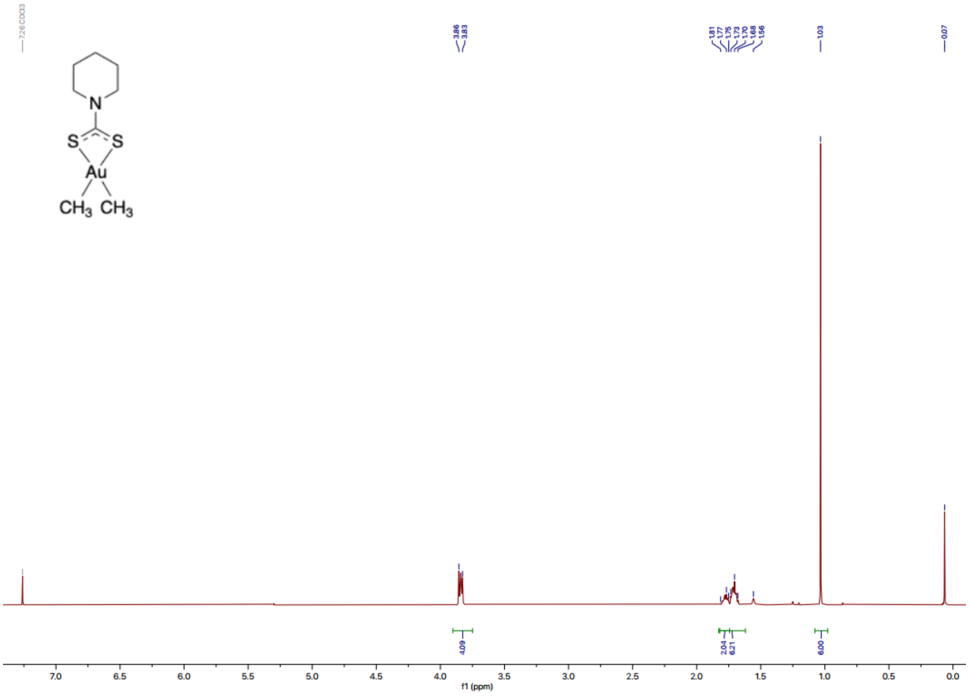

Figure S21: ^{1}H NMR spectrum of $[AuMe_2(pip\text{-}dtc)]$ in $CDCl_3$.

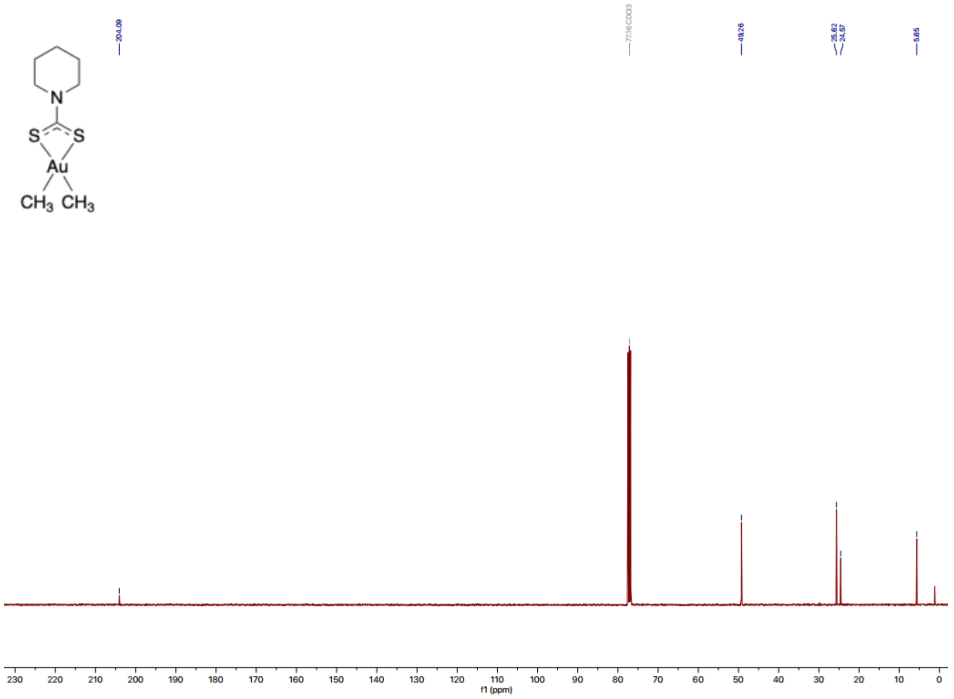

Figure S22: $^{13}C\{^{1}H\}$ NMR spectrum of $[AuMe_2(\text{pip-dtc})]$ in $CDCl_3$.

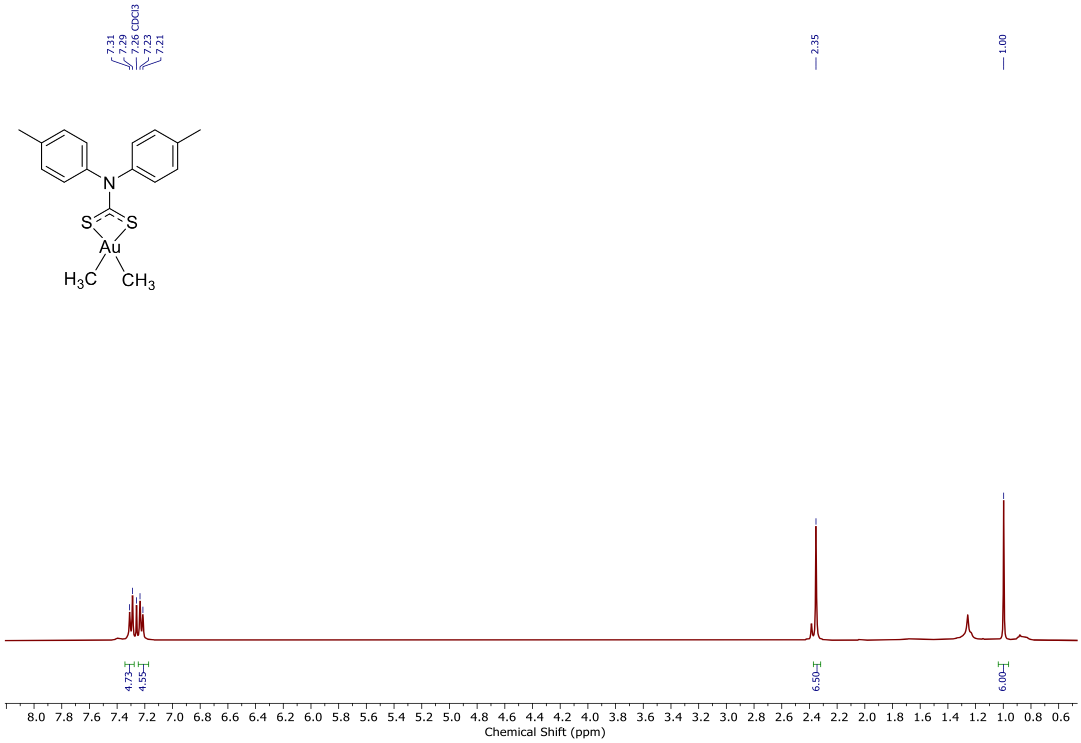

Figure S23: ^{1}H NMR spectrum of $[AuMe_2(p\text{-tolyl}_2\text{dtc})]$ in $CDCl_3$.

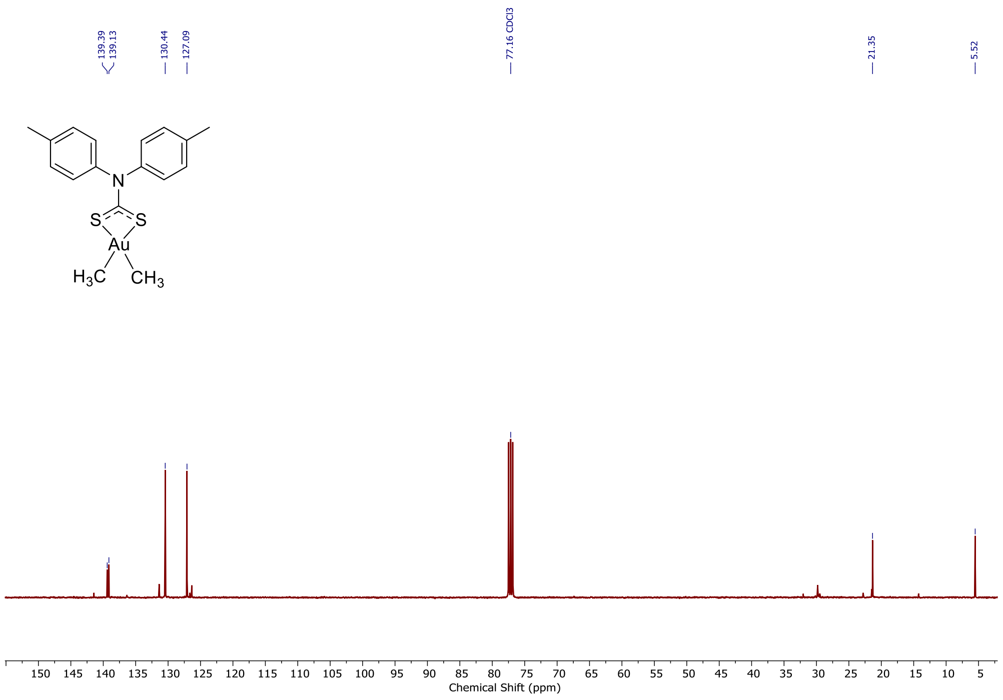

Figure S24: $^{13}C\{^{1}H\}$ NMR spectrum of [$AuMe_2$(*p*-tolyl$_2$dtc)] in $CDCl_3$.

5. References

S1) Hogarth, G. Metal-dithiocarbamate complexes: chemistry and biological activity. *Mini Rev. Med. Chem.*, **2012**, *12*, 1202–1215. DOI: 10.2174/138955712802762095.

S2) Carta, F.; Aggarwal, M.; Maresca, A.; Scozzafava, A.; McKenna, R.; Supuran, C. T. Dithiocarbamates: a new class of carbonic anhydrase inhibitors. Crystallographic and kinetic investigations. *Chem. Commun.*, **2012**, *48*, 1868–1870. DOI: 10.1039/C2CC16395K.

S3) Biswas, R.; Thakur, P.; Kaur, G.; Saha, S. Som. M.; Jhaihria, V.; Singh, H.; Ahmed, I.; Banerjee, B.; Chopra, D.; Sen, T.; Haldar, K. K. Interfacial Engineering of $CuCo_2S_4$/g-C_3N_4 Hybrid Nanorods for Efficient Oxygen Evolution Reaction. *Inorg. Chem.*, **2021**, *60*, 12355–12366. DOI: 10.1021/acs.inorgchem.1c01566.

S4) Mertens, R. T.; Parkin, S.; Awuah, S. G. Cancer cell-selective modulation of mitochondrial respiration and metabolism by potent organogold(III) dithiocarbamates. *Chem. Sci.*, **2020**, *11*, 10465–10482. DOI: 10.1039/D0SC03628E.

S5) Sarker, J. C.; Nash, R.; Boonrungsiman, S.; Pugh, D.; Hogarth, G. Diaryl dithiocarbamates: synthesis, oxidation to thiuram disulfides, Co(III) complexes $[Co(S_2CNAr_2)_3]$ and their use as single source precursors to CoS_2. *Dalton Trans.*, **2022**, *51*, 13061–13070. DOI: 10.1039/D2DT01767A.

S6) Brown, R. K.; Bunyan, J. N.; Agrawal, A.; Li, G.; Dautoras, D.; Sarker, J. C.; Keat, T. T.; Hicks, T.; Hogarth, G.; Pugh, D. A revised understanding of the speciation of gold(III) dithiocarbamate complexes in solution. *Dalton Trans.*, **2025**, *54*, 7627–7640. DOI: 10.1039/D5DT00240K.

S7) Uson, R.; Laguna, A.; Laguna, M.; Briggs, D. A.; Murray, H. H.; Fackler Jr., J. P. (Tetrahydrothiophene)Gold(I) or Gold(III) Complexes. *Inorg. Synth.*, **1989**, *26*, 86. DOI: 10.1002/9780470132579.ch17.

S8) Morgen, M.; Fabrowski, P.; Amtmann, E.; Gunkel, N.; Miller, A. K. Inclusion Complexes of Gold(I)-Dithiocarbamates with β-Cyclodextrin: A Journey from Drug Repurposing towards Drug Discovery. *Chem. Eur. J.*, **2021**, *27*, 12156–12165. DOI: 10.1002/chem.202101366.

S9) Viegers, T. P. A.; Trooster, J. M.; Bouten, P.; Rit, T. P. Mössbauer study of the dynamic behaviour of gold in molecular crystals. *J. Chem. Soc. Dalton Trans.*, **1977**, 2074–2080. DOI: 10.1039/DT9770002074.

S10) Forghieri, F.; Preti, C.; Tassi, L.; Tosi, G. Preparation, properties and reactivity of gold complexes with some heterocyclic dithiocarbamates as ligands. *Polyhedron*, **1988**, *7*, 1231–1237.

S11) Coles, S. J.; Allan, D. R.; Beavers, C. M.; Teat, S. J.; Holgate, S. J. W. Leading edge chemical crystallography service provision and its impact on crystallographic data science in the twenty-first century. In: Structure and Bonding, **2020**, Berlin, Heidelberg, Springer, 1–72.

S12) CrysAlisPRO, Oxford Diffraction/Agilent Technologies UK Ltd, Yarnton, England.

S13) Dolomanov, O. V.; Bourhis, L. J.; Gildea, R. J.; Howard, J. A. K.; Puschmann, H. *OLEX2*: a complete structure solution, refinement and analysis program. *J. Appl. Cryst.* **2009**, *42*, 339–341. DOI: 10.1107/S0021889808042726.

S14) Alvarez, S. A. A cartography of the van der Waals territories. *Dalton Trans.*, **2013**, *42*, 8617–8636. DOI: 10.1039/C3DT50599E.

S15) Stylianides, N.; Danopoulos, A. A.; Pugh, D.; Hancock, F.; Zanotti-Gerosa, A. Cyclometalated and Alkoxyphenyl-Substituted Palladium Imidazolin-2-ylidene Complexes. Synthetic, Structural, and Catalytic Studies. *Organometallics*, **2007**, *26*, 5627–5635. DOI: 10.1021/om700603d.
